# Paving the Way towards Sustainability of Polyurethanes: Synthesis and Properties of Terpene-Based Diisocyanate

**DOI:** 10.3390/molecules28207133

**Published:** 2023-10-17

**Authors:** Aliénor Delavarde, Sebastien Lemouzy, Aurélien Lebrun, Julien Pinaud, Sylvain Caillol

**Affiliations:** 1 ICGM, Univ Montpellier, CNRS, ENSCM, Montpellier, France; alienor.delavarde@enscm.fr (A.D.); sebastien.lemouzy@enscm.fr (S.L.); julien.pinaud@umontpellier.fr (J.P.); 2 IBMM, Univ Montpellier, CNRS, ENSCM, Montpellier, France; aurelien.lebrun@umontpellier.fr

**Keywords:** bio-based, polyurethanes, phosgene-free, isocyanate

## Abstract

Due to growing concerns about environmental issues and the decline of petroleum-based resources, the synthesis of new biobased compounds for the polymer industry has become a prominent and timely topic. P-menthane-1,8-diamine (PMDA) is a readily available compound synthesized from turpentine, a cheap mixture of natural compounds isolated from pine trees. PMDA has been extensively used for its biological activities, but it can also serve as a source of valuable monomers for the polymer industry. In this work, commercial PMDA (ca. 85% pure) was purified by salinization, crystallization, and alkali treatment and then converted into p-menthane-1,8-diisocyanate (PMDI) through a phosgene-free synthesis at room temperature. A thorough analytical study using NMR techniques (^1^H, ^13^C, ^13^C-^1^H HSQC, ^13^C-^1^H HMBC, and ^1^H-^1^H NOESY) enables the characterization of the cis-trans isomeric mixtures of both PMDA and PMDI. These structural studies allowed for a better understanding of the spatial configuration of both isomers. Then, the reactivity of PMDI with a primary alcohol (benzyl alcohol) was studied in the presence of nine different catalysts exhibiting different activation modes. Finally, the use of PMDI in the synthesis of polyurethanes was explored to demonstrate that PMDI can be employed as a new biobased alternative to petrochemical-based isocyanates such as isophorone diisocyanate (IPDI).

## 1. Introduction

Polyurethanes (PU) are industrially important polymers since they are ranked as the seventh most produced synthetic polymer, with a worldwide consumption valued at US$ 83.1 billion in 2022 [1]. Generally, PU is obtained by reacting a polyfunctional alcohol (polyol) with a polyfunctional isocyanate (polyisocyanate). Most of the time, these reactive compounds are derived from petroleum, a resource subject to shortage risks and price fluctuations. To address sustainability concerns regarding PU monomers, both academic and industrial teams have been working on the development of bio-derived polyols and polyisocyanates. Both sides successfully synthesized a variety of bio-based polyols from short bio-based diols/polyols [2,3,4] like bio-propanediol, bio-butanediol, glycerol, vegetable oil-derived polyols [5,6,7,8], lignin-derived polyols [9,10,11,12], and terpene-derived polyols [13] to polymer bio-based polyols [14,15,16]. However, only a few teams have worked on the synthesis of bio-based polyisocyanates, particularly by exploring the available phosgene-free methods. For example, Wadgaonkar and coworkers [17,18] highlighted the possibility of designing a toluene diisocyanate “look-alike” compound from cashew nutshell liquid (CNSL) using the well-known method of amine phosgenation with bis(trichloromethyl) carbonate (triphosgene). This research group [19] also used the Curtius rearrangement to design the 5,5′-diisocyanato-2,2′,3,3′-tetramethoxy-1,1′-biphenyl (BDI) from vanillic acid. Similarly, Klein et al. [20] reported the synthesis of furan-derived diisocyanates. However, in these examples, the methods used to synthesize isocyanates exhibit several disadvantages. In particular, the Curtius rearrangement requires the utilization of an acyl azide intermediate, which can raise handling and safety concerns. The thermolysis of urethane and the reductive carbonylation of nitro compounds have several drawbacks. Notably, the reductive carbonylation reaction necessitates a high pressure of carbon monoxide, a highly toxic gas. Both of these methods are energy-intensive and require elevated reaction temperatures exceeding 200 °C [21]. As we recently demonstrated for the synthesis of bisguaiacol F diisocyanate [22], these issues can be avoided by using a very promising and relatively under-exploited method reported by Knolker’s et al. [23] in the 1990s to synthesize bisguaiacol F diisocyanate. This phosgene-free and room-temperature protocol uses di-tert-butyl dicarbonate (Boc_2_O) in the presence of a catalytic amount of 4-dimethylaminopyridine (DMAP) to obtain isocyanate compounds from amines. The only limitation of this method is related to the choice of the starting material, as only poorly nucleophilic (e.g., aromatic and very sterically hindered alkyl) primary amines can be used to avoid the formation of urea side-products. Since p-menthane-1,8-diamine is composed of two very sterically congested primary amines, it is a good candidate for the preparation of its isocyanate analogue using Knolker’s protocol.

**Scheme 1:**
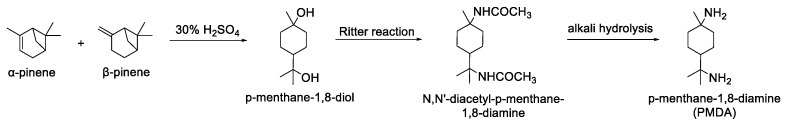
Synthesis of p-menthane diamine through Jians et al. [25] procedure.

P-menthane-1,8-diamine is derived from turpentine [24], a mixture of natural compounds isolated from pine trees. The main components of turpentine, α- and β-pinene, are considered the most abundant natural raw materials used for the synthesis of terpine hydrate in industrial production [25]. This monohydrate plays an essential role in the preparation of medicine intermediates, fragrances, and other fine chemicals like p-menthane-1,8-diamine (PMDA) [26]. Indeed, p-menthane-1,8-diamine, a primary alicyclic diamine, is widely used as an antibacterial agent, curing agent, or reactive raw material. Traditionally, PMDA is produced by the acidic hydrolysis of *N*,*N*′-diformacyl-p-menthane-1,8-diamine obtained from terpine hydrate and hydrogen cyanide [26]. However, this method induces the production of numerous by-products and uses hydrogen cyanide, a poisonous compound. Henceforth, PMDA is obtained via alkali hydrolysis of the *N*,*N*′-diacetyl-p-menthane-1,8-diamine amine moiety at high temperature (Scheme 1) [25,27].

We decided to work with commercially available PMDA and described, for the first time, a detailed purification protocol. Indeed, Zhu et al. [25] mentioned the possibility of purifying PMDA through salinization, crystallization, and alkali treatment, but no detailed experimental protocol was provided in their work. Afterwards, we synthesized PMDI via the phosgene-free, room-temperature route using Knolker’s et al. [23] procedure. 

Next, we carried out an analytical study of p-menthane diamine, diisocyanate, and dicarbamate using NMR techniques (^1^H, ^13^C, ^13^C-^1^H HSQC, ^13^C-^1^H HMBC, and ^1^H-^1^H NOESY). This investigation facilitated our understanding of the spatial arrangement of *cis* and *trans* isomers within the difunctionalized p-menthane-based compounds. In parallel, we explored the reaction between PMDI and a primary alcohol activated by nine different catalysts. Lastly, we designed a PMDI-based thermoset and compared its physicochemical properties as well as the reaction rate during material fabrication. Therefore, we believe that PMDI can be viewed as a biosourced substitute for the petrochemical-based monomer isophorone diisocyanate, given their structural similarities.

## 2. Results and Discussion

### 2.1. PMDI Synthesis

Commercially available p-menthane 1,8-diamine, with a purity of approximately 85%, was employed as the starting material. PMDA is sold as a clear to pale-yellow liquid. In order to ensure good repeatability and reproducibility of our experiments, it was necessary to enhance the purity of the initial diamine compound using a simple, efficient, and rapid purification method. We turned our attention to a three-step purification procedure to isolate PMDA. First, the salinization of crude PMDA with a solution of acetic acid allowed us to obtain protonated PMDA (PMDAH_2_^2+^)(2AcO^−^) as a white to yellowish solid. We then proceeded with the two-solvent cold crystallization step by dissolving the protonated diamine (PMDAH_2_^2+^)(2AcO^−^) in a minimal volume of isopropanol, followed by the addition of acetone. This process allowed us to obtain a solid product in a few hours of refrigeration at 3 °C. Finally, treating the purified (PMDAH_2_^2+^)(2AcO^−^) with a saturated aqueous solution of sodium hydroxide resulted in a pale-yellow liquid with a purity of approximately 99%, with yields in the 65–70% range. The purified PMDA was fully characterized by ^1^H, ^13^C, ^13^C-^1^H HSQC, ^13^C-^1^H HMBC, and ^1^H-^1^H NOESY, as well as by FTIR, GC-MS, and LC-MS analysis. The NMR chemical shifts of the purified PMDA are depicted in Table 1.

**Scheme 2:**
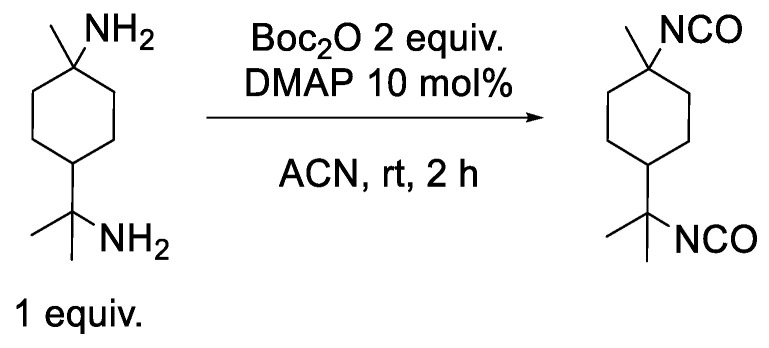
Synthesis of PMDI from PMDA under phosgene-free conditions.

We were then able to proceed with the synthesis of PMDI using phosgene-free and transition metal-free conditions. Building upon the work of Knolker [23] and our recent results [22], we obtained the corresponding diisocyanate from PMDA using a catalytic amount of DMAP (10 mol%) and a slight excess of di-*tert*-butyldicarbonate (2.2 equiv.) in dry acetonitrile. We monitored the reaction by GC-MS and reached full conversion of the diamine after 2 h. However, we observed residual amounts of DMAP and Boc_2_O. To address this issue, we isolated PMDI using flash column chromatography on silica gel. This purification step effectively removed DMAP residuals, although traces of di-*tert*-butyldicarbonate were still present and visible in GC-MS chromatograms. The adjustment of the flash column chromatography parameters did not prevent the presence of a residual amount of Boc_2_O in the isolated PMDI. We thus decided to reduce the initial amount of Boc_2_O until reaching strictly stoichiometric conditions (1 equiv. of PMDA for 2 equiv. of Boc_2_O), as explained in Scheme 2. Indeed, with stoichiometric conditions, we were able to achieve complete conversion of PMDA into PMDI within 2 h and fully consume the Boc_2_O reagent. 

PMDI was obtained as a light yellow to colorless liquid with a purity above 99% and a yield of 75%. The isolated PMDI was fully characterized by ^1^H, ^13^C, ^13^C-^1^H HSQC, ^13^C-^1^H HMBC, and ^1^H-^1^H NOESY, as well as FTIR, GC-MS, and LC-MS analysis. The NMR chemical shifts of the obtained PMDI are exposed in Table 1. Moreover, back-titration of NCO functions was carried out to confirm the functionality and purity of PMDI. To perform the volumetric determination, we initially reacted PMDI with an excess of amine and then titrated the remaining unreacted amine with a hydrochloric acid solution. To this end, we employed di-n-butylamine, a highly reactive amine, and a solution of bromocresol green for colorimetric titration. The titration was repeated three times, allowing us to obtain an experimental NCO value of 37.71%, while the expected theoretical NCO value was around 37.85%. This observed NCO content shows that PMDI was efficiently purified, and it is consistent with the result obtained by GC-MS analysis.

### 2.2. NMR Characterization of PMDA, PMDI, and p-Menthane-1,8-Dicarbamate

A comprehensive characterization of purified PMDA and PMDI was conducted by NMR analysis to elucidate the spatial configuration of *cis* and *trans* isomers. Indeed, as indicated by the GC-MS chromatograms, PMDA and PMDI exhibit two distinct peaks corresponding to either cis or trans isomers. However, it is impossible to attribute one peak to a particular isomer using GC-MS. Nonetheless, NMR was employed as an effective analytical method for differentiating the *cis* and *trans* isomers, and consequently the major and minor isomers. This was made possible by combining the PMDA and PMDI NMR studies with the NMR study of p-menthane dicarbamate (PMDC).

PMDI possesses a unique chemical structure where no hydrogen atoms are in the α position of the isocyanate group. In order to track the disappearance of the isocyanate groups and the formation of carbamate groups using ^1^H NMR analysis, it is thus crucial to employ an alcohol with hydrogen atoms that do not overlap with the ^1^H NMR signals of PMDI. Therefore, we chose to work with benzyl alcohol, which features reactive primary alcohol functionality in the benzylic position, and we used the two benzylic protons as a probe for the monitoring of the carbamate formation. Since the hydrogen atoms in the α-position of the alcohol are attached to a six-membered aromatic ring, we were confident that there would be no interference when monitoring the signals corresponding to the CH_2_-OCONH moiety. 

This NMR study enabled the distinction between major and minor isomers of PMDI. Specifically, to differentiate between the positions of both CH_2_ benzyl groups (position 1 or 8 in the molecule), NOE correlations (Figures S16 and S17) were observed between methyls and NH, and subsequently between NH and the methylene groups. For the major cis-isomer, the methyl group C*H*_3_(7) at 1.33 ppm shows a correlation with the N*H*(1) function at 4.53 ppm. The latter exhibits a correlation with methylene C*H*_2_ at 5.06 ppm. For trans-isomer, methyl groups 9 and 10 (1.27 ppm) correlate with N*H*(8) at 4.67 ppm, and this N*H*(8) correlates with C*H*_2_ at 5.04 ppm. Furthermore, the configuration of position 1 was established thanks to additional NOE correlations. The axial C*H*(4) of the cis-isomer at 1.87 ppm correlates with H_ax_(2/6) at 1.28ppm and H_ax_(3/5) at 1.55ppm. The N*H*(1) shows correlations with H_eq_(2/6) at 2.17 ppm and H_eq_(3/5) at 1.15 ppm, confirming the axial position of C*H*_3_(7).

**Figure 1:**
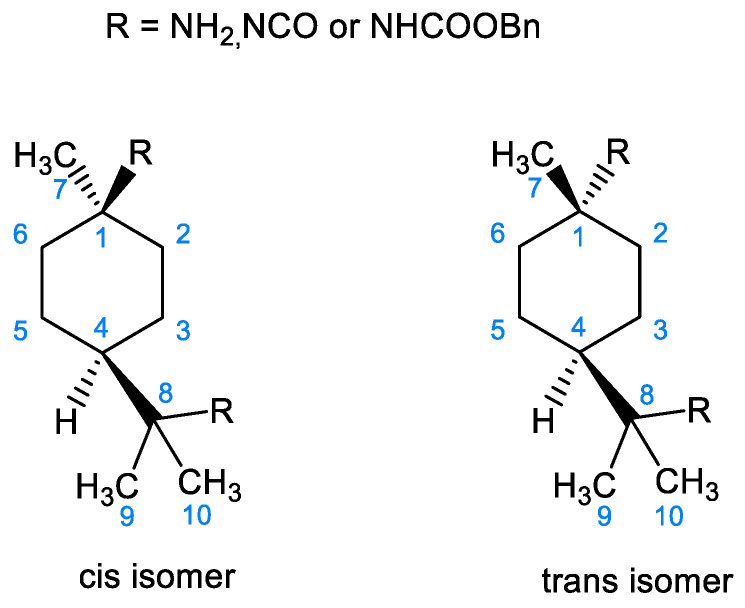
Spatial representation of the cis and trans isomers of p-menthane diamine, diisocyanate, and dicarbamate components.

These interpretations were confirmed by the literature [28], where it was demonstrated that the signal of the *C*H_3_(7) methyl group in ^13^C NMR undergoes an upfield shift of approximately 6 ppm when it is in the equatorial orientation compared to the axial orientation. Consequently, the major isomer can be identified as the *cis* isomer with an axial orientation of the *C*H_3_(7) and an equatorial orientation of the amine, isocyanate, or carbamate group, while the minor isomer can be identified as the trans-isomer. This study also presents, for the first time, a comprehensive description of the chemical shifts and spatial positioning of the various atoms of PMDA, PMDI, and PMDC molecules, as illustrated in Figure 1, Table 1 and Table 2.

**Table 1:** ^1^H NMR chemical shift of PMDA, PMDI and PMDC.

^1^H	R = NH_2_ (Purified)		R = NCO		R = NHCOOBn	
	*cis*	*trans*	*cis*	*trans*	*cis*	*trans*
**2/6 ax**	1.34	1.27	1.37	1.63	1.28	1.52
**2/6 eq**	1.51	1.61	1.85	1.89	2.17	2.00
**3/5 ax**	1.62	1.68	1.72	1.76	1.55	1.65
**3/5 eq**	1.25	1.12	1.39	1.22	1.15	1.19
**4**	1.04	1.10	1.28	1.30	1.87	1.86
**7**	1.08	1.05	1.36	1.34	1.33	1.34
**9/10**	1.04	1.04	1.33	1.32	1.25	1.27
**1-NH**					4.53	4.75
**8-NH**					4.67	4.68
**1-CH_2_ (Bn)**					5.06	nd
**8-CH_2_ (Bn)**					5.04	nd

nd = not determined. The background color is here to indicate that there is no signal attributed to the proton of the corresponding molecule.

**Table 2:** ^13^C NMR chemical shift of PMDA, PMDI and PMDC.

^13^C	R = NH_2_ (Purified)		R = NCO		R = NHCOOBn	
	*cis*	*trans*	*cis*	*trans*	*cis*	*trans*
**1**	47.8	49.0	58.1	57.6	52.0	52.6
**2/6**	40.1	41.9	39.5	40.1	36.8	37.3
**3/5**	22.8	24.5	23.4	24.0	22.6	23.4
**4**	49.1	49.2	47.8	47.5	44.3	44.9
**7**	33.1	25.7	31.6	25.5	28.0	21.9
**8**	51.3	51.2	61.0	60.7	55.5	55.5
**9/10**	28.5	28.5	28.1	28.5	24.5	24.5
**1-NCO**			122.2	122.6/122.8	154.7	nd
**8-NCO**			122.5		154.6	nd
**CH_2_ (Bn)**					66.1	nd
**C_q_ (Bn)**					136.9	nd

nd = not determined. The background color is here to indicate that there is no signal attributed to the proton of the corresponding molecule.

**Figure 2:**
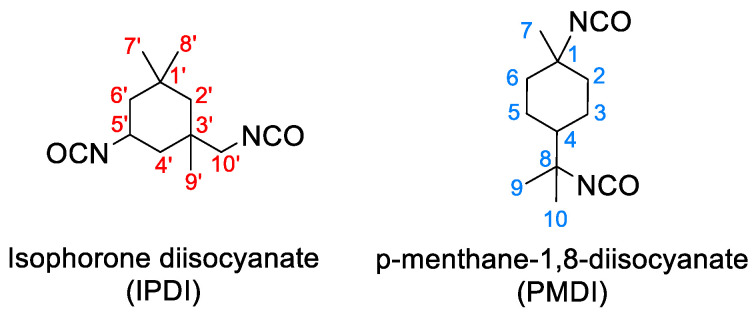
Chemical structures of IPDI and PMDI compounds.

PMDI is a molecule that exhibits structural similarities to the well-known isophorone diisocyanate compound. Both diisocyanates, PMDI and IPDI, are six-membered heterocyclic, non-aromatic compounds (Figure 2). They are also asymmetrical molecules with non-equivalent isocyanate groups. Specifically, PMDI has a quaternary cycloaliphatic isocyanate group on C_1_ and a quaternary isocyanate group on C_8_, while IPDI features a tertiary cycloaliphatic isocyanate group on C_5′_ and a secondary isocyanate group on C_10′_ [29]. IPDI is a monomer used to synthesize polyurethane systems for various industrial applications. IPDI is widely used in the formulation of coatings and acts as a hardener and cross-linking agent, generating durable and fast-drying coatings. It is also used in the manufacturing of adhesives as it provides excellent adhesion to a variety of substrates, including metals, woods, plastics, and composites [29,30,31,32]. IPDI can also be used in the production of thermoplastic polyurethanes and elastomers, which are used in several applications, such as automotive, electronics, and consumer products. To the best of our knowledge, there is currently no bio-based alternative to IPDI available on the market, despite its wide-ranging applications in various fields.

Given their similar structures, a comparison was made between the reactivity of PMDI and IPDI using NMR analyses. Moreover, an investigation was carried out to assess the influence of catalyst type on the reactivity of PMDI.

### 2.3. Influence of Catalyst Nature on the Reactivity of PMDI

**Scheme 3:**
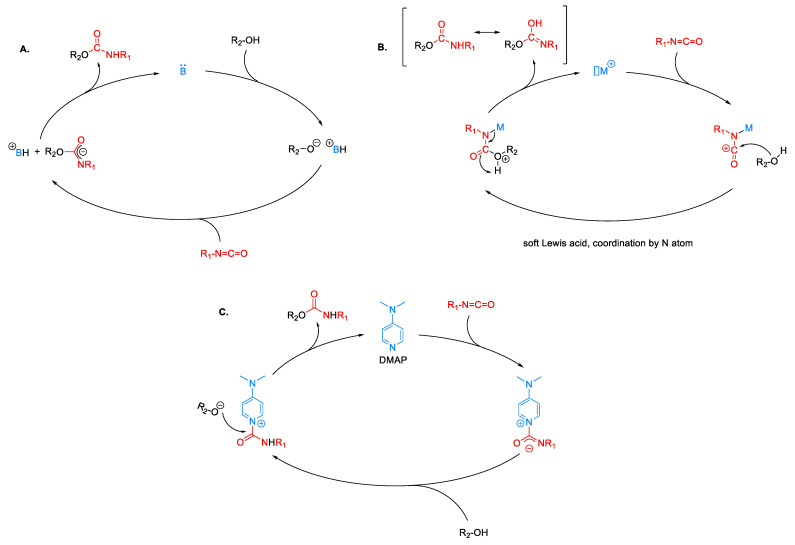
Activation modes of the catalyzed isocyanate-alcohol reaction [34] with Brønsted bases [35] (**A**), Lewis acids [36] (**B**), and relay nucleophiles [37,38,39] (**C**).

**Figure 3:**
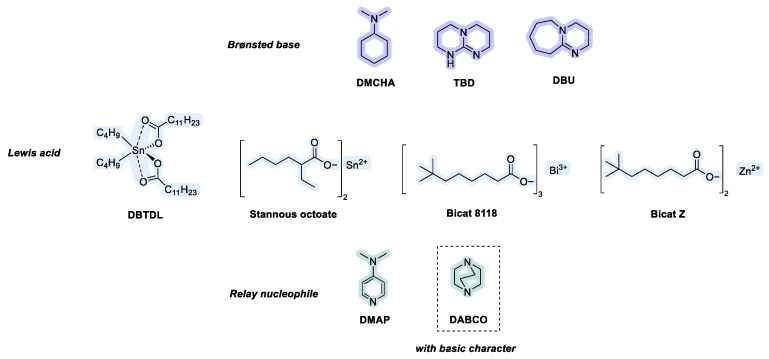
Structure of the catalysts employed for the activation of the isocyanate-alcohol reaction, their abbreviations, and their categorization types.

The catalytic activation of the isocyanate-alcohol reaction, in our case PMDI and benzyl alcohol, was investigated using a range of catalysts with three different activation modes, as seen in Scheme 3. In total, nine catalysts (Figure 3) *N*,*N*-dimethylcyclohexylamine (DMCHA), 1,5,7-triazabicyclo [4.4.0]dec-5-ene (TBD), 1,8-diazabicyclo [5.4.0]undéc-7-ène (DBU), dibutyl tin dilaurate (DBTDL), stannous octoate, bismuth decanoate (Bicat 8118), zinc decanoate (Bicat Z), 4-dimethylaminopyridine (DMAP), and 1,4-diazabicyclo [2.2.2]octane (DABCO) of Brønsted base, Lewis acid, or relay nucleophile types were employed. In the case of the alcohol-isocyanate reaction catalyzed by a Lewis acid, two coordination modes are potentially achievable, allowing the binding of the metal center either to the oxygen or to the nitrogen atom. Indeed, soft Lewis acids have a propensity to readily coordinate with atoms or groups of atoms that possess lower electron densities and greater polarizabilities, such as nitrogen atoms. On the contrary, oxygen is more electronegative and has a greater affinity for electrons than nitrogen, making it “harder”. Nitrogen is thus considered relatively “softer” than oxygen. However, it is important to note that this classification is a simplification, and the hardness or softness of atoms can vary based on the chemical context and specific interactions. In Scheme 3B, an activation mode involving coordination by the nitrogen atom with a soft Lewis acid catalyst is proposed. Nevertheless, achieving coordination by the oxygen atom is also potentially feasible when harder Lewis acid catalysts are used [33].

As explained previously, PMDI has two alpha-quaternary isocyanate groups, including one attached to the cycloaliphatic ring. Differentiating the reactivity between the two isocyanate groups is challenging as they are both quaternary, which prevents us from following the consumption of one isocyanate over the other one by ^1^H NMR spectroscopy. However, the isocyanate group on C_1_ appears to be less hindered than that on C_8_, which could enhance its reactivity. Indeed, it has been shown that in most instances, the secondary isocyanate group in IPDI exhibits higher reactivity due to lower steric hindrance in the chair conformation when compared to the quaternary isocyanate group [40,41]. Nevertheless, some catalysts have proven to be effective for activating both isocyanates in a similar manner [41,42,43,44,45,46,47,48]. For example, DABCO slightly enhances the reactivity towards the quaternary isocyanate group of IPDI and even results in equal reactivity for both isocyanate groups [40]. However, the study of the different reactivities of the isocyanate groups of IPDI is limited to a few catalysts. Our strategy was to work with the most conventional and commonly used catalysts to enhance the isocyanate-alcohol reaction and compare their effects on PMDI reactivity. However, we were not able to distinctly follow the reactivity of the quaternary isocyanate carried by the cycloaliphatic ring and the one carried by C_8_ by NMR analysis. We were limited by the overall reactivity of PMDI with respect to the alcohol-bearing components and thus were unable to differentiate the intrinsic reactivities of each isocyanate group within the PMDI structure.

**Scheme 4:**
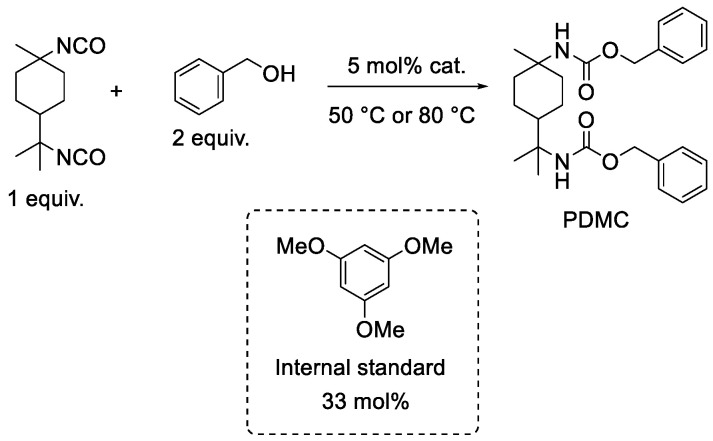
Synthesis of PMDC with 1,2,5-trimethoxybenzophenone as an internal standard.

To follow the isocyanate-alcohol reaction kinetics through ^1^H NMR analysis, we employed benzyl alcohol as a mono-alcohol to react with IPDI in the presence of an internal standard in deuterated toluene. We used a 5 mol% catalyst loading at two distinct temperatures (80 °C and 50 °C), as seen in Scheme 4. In fact, the reactivity of some catalysts at 80 °C was so high that the reaction took place instantaneously before we could even proceed to NMR monitoring, redirecting us towards the lower temperature of 50 °C.

**Figure 4:**
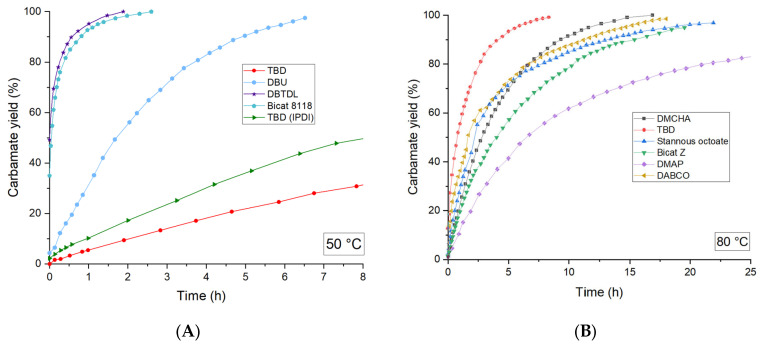
Influence of catalysts on the kinetics of the reaction between PMDI and benzyl alcohol at 50 °C (**A**) and 80 °C (**B**).

Bi- and Sn-based catalysts exhibit the highest catalytic activity. Indeed, we had to lower the temperature from 80 °C to 50 °C to be able to clearly monitor the carbamate formation. The reactivity of DBTDL and Bicat 8118 is so strong that the first kinetic points obtained, when following the reaction kinetics by NMR analysis, had already reached 45% and 35% of conversion, respectively, for DBTDL and Bicat 8118, as seen in Figure 4.

Amidine and guanidine catalysts, namely DBU and TBD, and a tertiary amine catalyst, DMCHA, exhibited the strongest catalytic activity after Sn- and Bi-based Lewis acid catalysts. However, the catalytic activity of TBD and DMCHA cannot challenge DBTDL, Bicat 8118, and DBU catalytic activity, as DBTDL achieved complete conversion of the isocyanate groups into carbamate groups after 1 h 50 min, Bicat 8118 after 2 h 35 min, and DBU after 6 h 30 min at 50 °C. Comparatively to DBTDL, TBD only achieved 9% conversion after 1 h 50 min in the same conditions (50 °C), as seen in Figure 4. Nevertheless, at 80 °C, the TBD-catalyzed reaction reached full conversion after 8 h and 20 min of reaction, whereas DMCHA provided the same result in nearly 17 h. DMAP, an organic catalyst with a relay nucleophile activation mode, exhibited the lowest catalytic activity. Even after 40 h of reaction, complete conversion of the isocyanate function could not be achieved. Stannous octoate, Bicat Z, and DABCO demonstrated comparable catalytic activities. They were able to achieve full conversion within approximately 20 h at 80 °C. Furthermore, we compared the reactivities of PMDI and IPDI, both activated with 5 mol% of TBD, at 50 °C and 80 °C. As anticipated, at 50 °C, the reactivity of IPDI was slightly higher than that of PMDI in the same conditions. However, at 80 °C, their reactivities were comparable. Indeed, it was expected that IPDI would exhibit a higher reactivity than PMDI, given that IPDI has fewer hindered isocyanate groups and also possesses an α-secondary isocyanate. Nevertheless, our results strongly suggest that during polymer elaboration, IPDI-based formulations have better reactivities than PMDI-based formulations. 

The above-mentioned studies have allowed us to classify the reactivities of each catalyst and identify the one with the greatest capacity for activating the reaction. In general, hard Lewis acid catalysts exhibit the strongest reactivity, and among them, DBTDL showcases the most potent catalytic effect. Brønsted base catalysts provide an alternative to transition-metal catalysts as they demonstrate favorable reactivity, which can be particularly appealing for applications that require metal-free formulations, such as in the electronic, food, cosmetics, and pharmaceutical industries.

### 2.4. Synthesis, Characterization, and Reaction Study of PMDI- and IPDI-Based Polyurethanes

Due to the common usage of IPDI in coating formulations, our objective was to synthesize PU thermoset materials from IPDI and PMDI and to compare their reactivities and properties. In order to achieve partially bio-sourced PU materials, we utilized two polyols derived entirely from renewable resources, namely poly(1,3-propanediol) and glycerol. Specifically, the PMDI monomer possesses a bio-based content of 83%, and the IPDI monomer is fully derived from petroleum resources. By employing a two-step method to develop a PMDI-based polyurethane, we successfully obtained a PU material with a remarkable biobased content of 92%. 

**Scheme 5:**
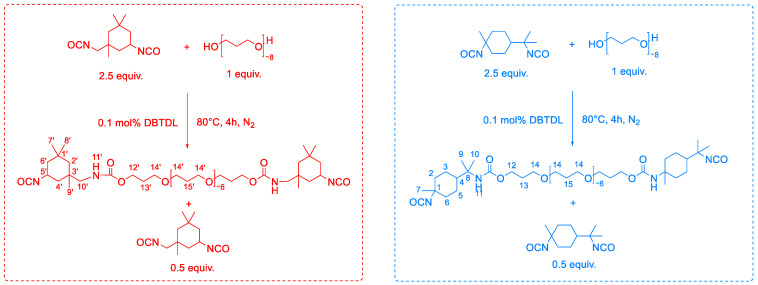
Idealized synthesis of NCO-terminated prepolymer with IPDI (in red) and PMDI (in blue).

Firstly, we prepared an NCO-telechelic prepolymer at 80 °C by reacting the diisocyanate compound (IPDI or PMDI) with the poly(1,3-propanediol) with a molar mass of approximately 500 g.mol^−1^ (marketed under the trade name Velvetol^®^ H500, WeylChem International GmbH, Frankfurt am Main, Germany) in the absence of a catalyst. The conversion of the reaction was assessed by monitoring the disappearance of the signal corresponding to the two CH_2_ close to the terminal alcohol groups of Velvetol^®^ H500 at 3.50 ppm (t) and 3.59 ppm (t). Unfortunately, with the PMDI monomer, even after 4 h of reaction, the signal attributed to the terminal alcohol groups was still present in the ^1^H NMR spectra of the reaction aliquots, unlike for the preparation of the IPDI prepolymer. Moreover, the obtained integration values suggest that an oligomer was obtained instead of a prepolymer terminated by one PMDI unit. It is likely that there was an uneven addition of Velvetol^®^ H500 onto PMDI due to the slow reaction between the alcohol and isocyanate groups. Consequently, we used a relatively low amount of DBTDL (0.1 mol%) in both prepolymer syntheses to ensure a fast and efficient reaction between the NCO of PMDI or IPDI and the alcohol end groups of Velvetol^®^ H500. We were finally able to obtain the desired NCO telechelic prepolymers consisting of a single unit of Velvetol^®^ H500 combined with both diisocyanate monomers as displayed in Scheme 5.

**Figure 5:**
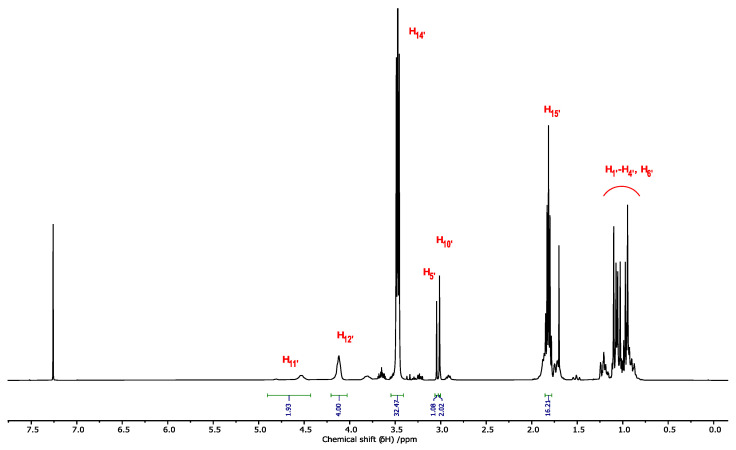
^1^H NMR spectrum of IPDI-based prepolymer.

**Figure 6:**
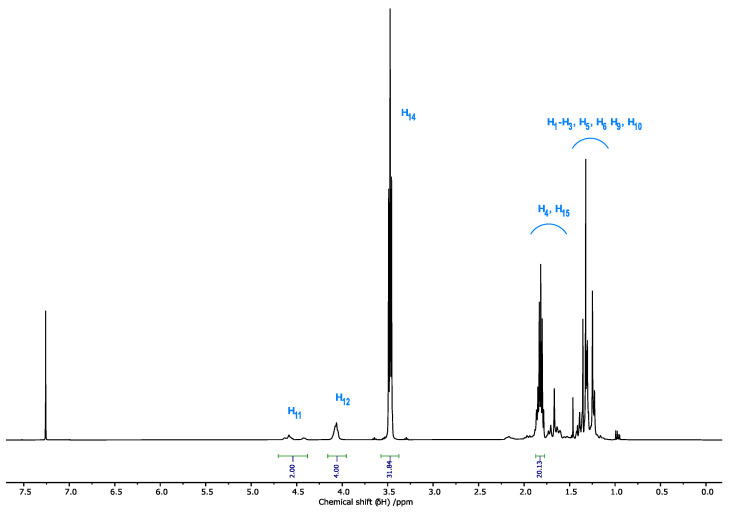
^1^H NMR spectrum of PMDI-based prepolymer.

The prepolymer structures were confirmed by ^1^H NMR, as seen in Figure 5 and Figure 6, ^13^C NMR, and FTIR analyses, as seen in Supplementary Information.

**Figure 7:**
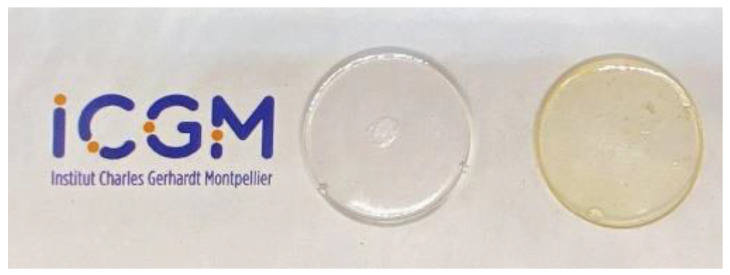
Photograph of IPDI-based thermoset (left) and PMDI-based thermoset (right).

Subsequently, the synthesized prepolymer was reacted with glycerol in a stoichiometric ratio with respect to the NCO functionality (NCO:OH = 1). The mixture was then stirred and cured in a silicon mold at 80 °C for 24 h. As seen in Figure 7, the IPDI-based thermoset is transparent and colorless, while the PMDI-based thermoset is transparent with a slight yellowish tint.

We characterized these two materials using FTIR, TGA, and DSC analyses and determined their swelling index and gel content. Additionally, to underscore and compare the reactivity of IPDI and PMDI monomers, we monitored the disappearance of the NCO group during thermoset formation at 80 °C using FTIR and measured the gelation time of both materials through rheological analysis using a multi-frequency method and a plate-plate geometry at 90 °C with a stress of 3 Pa.

Indeed, the intersection of G′(t) and G″(t) allows us to determine the gelation time of thermoset systems. More precisely, the point where G′(t) intersects with G″(t) corresponds to the moment where the system undergoes a transitional phase. This transition is associated with the progression from an “elastic-dominated” formulation (a liquid) to a “viscosity-dominated” formulation, corresponding to the initial stage of a crosslinked/solid material. Table 3 presents the gelation time for three different frequencies under a stress of 3 Pa, along with the average gelation time obtained for both formulations. Additionally, the G′ and G″ curves for both formulations at the three different frequencies are displayed in Figures S25 and S27. The IPDI-based formulation has an average gelation time of 430 s, corresponding to approximately 9 min, while the PMDI-based formulation has an average gelation time of 4875 s, corresponding to 1 h 23 min. These results highlight the higher reactivity of IPDI compared to PMDI, in agreement with what was observed in a diluted system during the synthesis of the prepolymer. The gelation of the IPDI-based formulation is eleven times faster than the PMDI-based formulation gelation time. It was also predictable considering the steric hindrance between the NCO groups of the two monomers, as PMDI has a higher hindrance in its NCO groups compared to IPDI.

**Figure 8:**
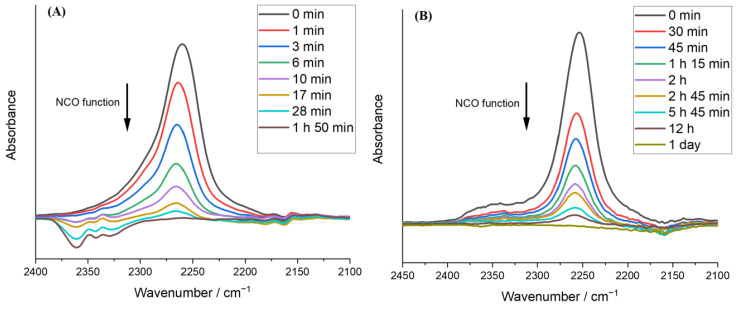
Evolution of the C=O stretching peak of the NCO function of IPDI-based formulation (**A**) and PMDI-based formulation (**B**) over time at 80 °C.

In parallel, we monitored the disappearance of the C=O signal of the NCO group at 2260 cm^−1^ during the curing process of the formulation at 90 °C by using a Nicolet 210 Fourier transform infrared (FTIR) spectrometer equipped with a Specac golden gate attenuated total reflection (ATR) heating cell. The degree of conversion for the curing reaction can be expressed as U = (A_0_ − A_t_)/A_0_, where A_0_ represents the initial peak area of the C=O stretching (OCN function) at t_0_ and A_t_ represents the peak area of the C=O stretching (OCN function) at a given time. In accordance with the gelation time results, the complete conversion of isocyanate into carbamate groups was faster with the IPDI monomer compared to the PMDI monomer. Specifically, IPDI-based formulations achieved 80% conversion after 6 min, whereas PMDI-based formulations reached the same level of conversion after 1 h 28 min of reaction. Furthermore, the complete disappearance of NCO peaks took a significantly longer time for PMDI monomer (24 h in total), while it was considerably faster for the IPDI-based formulation (1 h 50 min), as seen in Figure 8.

**Figure 9:**
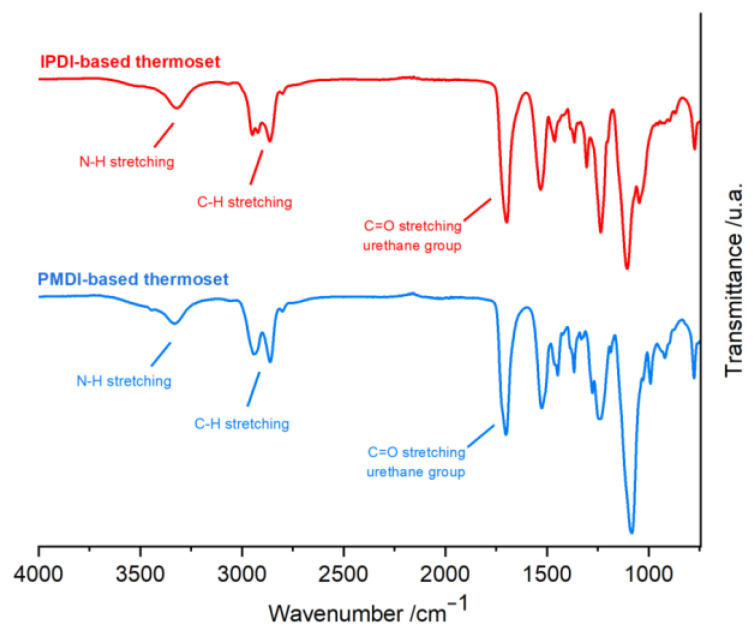
FTIR spectrum of IPDI- and PMDI-based thermosets.

Figure 9 shows the FTIR spectra of cured materials obtained with IPDI (top) and PMDI (bottom) monomers. In both cases, the spectra display a broad band ranging from 3660 to 3200 cm^−1^ that corresponds to the N-H bond of the urethane groups. Additionally, both spectra exhibit a distinct peak at around 1700 cm^−1^, indicating the presence of the C=O functionality of the urethane groups. Furthermore, the absence of a band around 2300 to 2200 cm^−1^ corresponding to the C=O stretching of the isocyanate bond indicates the complete consumption of the isocyanate groups for both thermosets.

**Figure 10:**
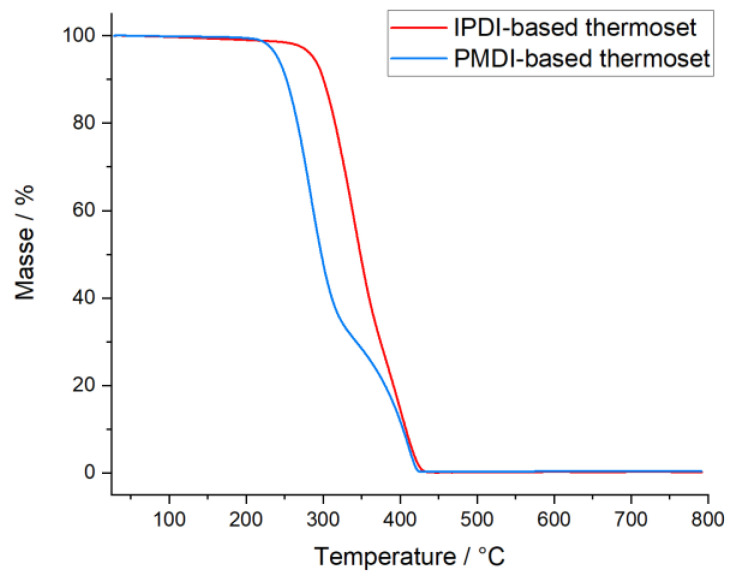
TGA curves of IPDI- and PMDI-based thermosets.

Finally, the thermal stability of both cured materials was studied by TGA analysis under a nitrogen atmosphere (Table 4). While both materials have the same maximal temperatures of degradation around 430 °C, it appears that the PMDI-based material displays a slightly different thermal behavior. More specifically, the thermogram of PMDI-based material exhibits two distinct mass losses, as shown in Table 4 and Figure 10, which can be explained by the presence of a weaker linkage in the PMDI-based system.

**Figure 11:**
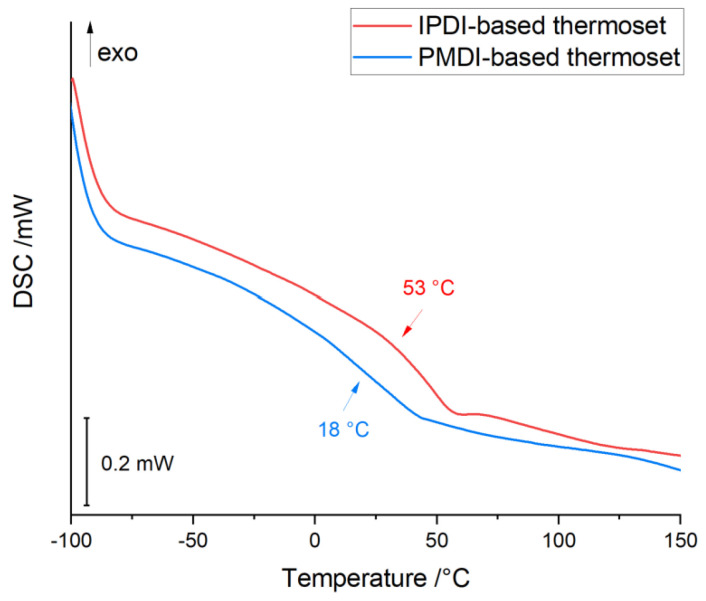
DSC curves of IPDI- and PMDI-based thermosets.

The steric hindrance of PMDI isocyanate groups reduces its reactivity compared to IPDI, but it also appears to contribute to the creation of a weak point in the PMDI-derived polymer system. Regarding their characterization by DSC, both materials exhibit a glass transition, which is typical for thermoset materials. The IPDI-based thermoset has a *T_g_* value of 53 °C, while the PMDI-based thermoset has a *T_g_* value of 18 °C (Table 4 and Figure 11). These results are consistent with the behavior of the materials in the THF solvent. Specifically, the IPDI-based material has a swelling index of 50% at room temperature in THF, whereas the PMDI-based material has a swelling index of 75%. These results are closely related to the network size. The formation of the IPDI-based thermoset likely resulted in a narrower network compared to the PMDI-based thermoset. However, both materials exhibit a high gel content, indicating the formation of a fully crosslinked material. 

This indicates that, despite the lower reactivity of the PMDI monomer and its higher steric hindrance compared to IPDI, it is possible to employ PMDI in the design of polyurethane systems that showcase nearly identical physical and chemical attributes to IPDI-based materials. Furthermore, as PMDI originates from renewable resources, it offers a sustainable approach to polyurethane design.

## 3. Materials and Methods

### 3.1. General Information

P-menthane-1,8-diamine mixture of cis and trans isomers (PMDA, 85%), acetic acid (99.5%), potassium iodide (≥85%), di-*tert*-butyl dicarbonate (Boc_2_O, 99%), 4-(dimethylamino)pyridine (DMAP, ≥99%), 1,3,5-trimethoxybenzene (≥99%), isophorone diisocyanate mixture of cis and trans isomers (IPDI, ≥99%), d-chloroform (CDCl_3_, 99.5% D), were supplied by Sigma-Aldrich (Darmstadt, Germany) and used as received. D-toluene (99.5% D) was supplied by Fluorochem (Hadfield, UK) and used as received. Velvetol^®^ H500 was kindly supplied by WeylChem International GmbH (Frankfurt am Main, Germany) and used as received. Glycerol (≥99.5%) was purchased by Prolabo (Paris, France) and used as received. 

NMR spectra were recorded at 298 K on a Bruker Avance III 600 MHz NMR spectrometer using TCI Cryoprobe Prodigy^®^ (Bruker Biospin, Fällanden, Switzerland). Chemical shift data are given in δ ppm calibrated with residual protic solvent (e.g., CDCl_3_: 7.26 ppm − ^1^H/77.16 ppm − ^13^C). A ^13^C UDEFT sequence was used with a spectral width of 33,300 Hz and 4096 scans. In addition, 2D homonuclear ^1^H-^1^H g-COSY (1 scan, 512 real (t1) × 2048 (t2) complex data points) and 2D heteronuclear spectra of ^13^C-^1^H g-edited HSQC and ^13^C-^1^H g-HMBC were acquired to assign the compound (8 scans, 512 real (t1) × 2048 (t2) complex data points). Homonuclear 2D spectra ^1^H-^1^H NOESY were recorded using data matrices of 256 real (t1) × 2048 (t2) complex data points; 16 scans per t1 increment with a 3s recovery delay and a spectral width of 7200 Hz in both dimensions were used. Spectra were processed and visualized with Topspin 3.6.2 (Bruker Biospin) on a Linux station. Fourier transform infrared spectra were collected with a Thermo Scientific (Waltham, MA, USA) Nicolet 210 FT-IR spectrometer featuring a Specac Golden Gate attenuated total reflection (ATR) heating cell. High-resolution mass spectrometry (HRMS) data were acquired using an LC-TOF mass spectrometer (micrOTOF-Q) with electrospray ionization (ESI) in either positive or negative mode. GCMS analyses were conducted using electrospray ionization (ESI) on a Chimadzu QP2010SE mass spectrometer. Thermogravimetric analyses (TGA) of the cured polyurethanes were carried out on a Netzsch (Burlington, MA, USA) STA 449 F1 TGA. Nitrogen gas at a flow rate of 20 mL·min^−1^ was used as the protective gas. About 10 mg of the sample was placed in an alumina crucible and heated from room temperature to 800 °C with a heating ramp of 10 °C·min^−1^. Differential scanning calorimetry (DSC) analyses were performed using a Netzsch DSC 3500 Sirius calorimeter. Nitrogen gas at 40 mL·min^−1^ was used as the purge gas. Approximately 10 mg of the sample was placed in pierced aluminum pans. The thermal properties of the thermoset materials were examined between −100 and 200 °C at a heating rate of 20 °C·min^−1^ to observe the glass transition temperature. To determine gelation times, a Thermo Fischer Mars 60 rheometer was used with a plate-plate aluminum disposable geometry (25 mm diameter, 0.4 mm gap, 0.2 mL of formulation). Measurements were conducted at 80 °C with a stress of 3 Pa, employing a multi-frequency program with frequencies of 5 Hz, 7.10 Hz, and 10 Hz.

### 3.2. Experimental Section

#### 3.2.1. General Procedure for the Purification of p-Menthane-1,8-diamine (PMDA)

A solution of acetic acid (2.5 equivalents, 0.293 mol, 17.60 g) in 40 mL of dichloromethane was prepared in a 100 mL beaker and then poured into a round-bottomed flask containing 20 g of PMDA (1 equivalent, 0.117 mol). The resulting solid was washed three times with dichloromethane and concentrated under reduced pressure. The solid was purified through cold recrystallization. Initially, a small amount of isopropanol was used to dissolve the solid, and then acetone was added to enable the recrystallization of the product. The solid was filtered using a Büchner funnel, followed by an alkali treatment process in which PMDAH^+^ solids were dissolved using a sodium hydroxide-saturated aqueous solution. The aqueous phase was extracted three times with dichloromethane, washed once with a saturated brine solution, and finally dried over MgSO_4_. The product was concentrated under reduced pressure and dried. A highly pure (≥99%) p-menthane-1,8-diamine was obtained as a yellowish liquid with a yield of 70%.

#### 3.2.2. General Procedure for the Synthesis of p-Menthane-1,8-diisocyanate (PMDI)

A solution of di-tert-butyl dicarbonate (2.0 equivalents, 11.74 mmol, 2.56 g) and DMAP (0.1 equivalent, 0.59 mmol, 0.072 g) in 10 mL of dry acetonitrile was prepared in a 50 mL round-bottomed flask with a magnetic stirrer. Subsequently, a solution of p-menthane-1,8-diamine (1 equivalent, 5.87 mmol, 1 g) in 10 mL of dry acetonitrile was gradually added to the previous solution. The resulting mixture was stirred at room temperature for 2 h and then concentrated under reduced pressure. The crude product was subjected to purification using flash column chromatography on silica gel with cyclohexane/ethyl acetate as eluents. After solvent evaporation, a highly pure (≥99%) p-menthane-1,8-diisocyanate was obtained as a white to yellowish liquid with a yield of 75%.

#### 3.2.3. General Procedure for the Synthesis of p-Menthane-1,8-dicarbamate Monitoring by ^1^H NMR

In a test tube, 35 mg of 1,3,5-trimethoxybenzene (33 mol% relative to the diisocyanate, 0.21 mmol), a catalyst (5 mol% relative to the diisocyanate), 0.146 g of benzyl alcohol (2 equivalents, 1.26 mmol), and 0.150 g of diisocyanate (1 equivalent, 0.68 mmol) were introduced in this specific order. The test tube was preserved in an ice bath, and 0.4 mL of deuterated toluene (toluene-d_8_) was added. The solution obtained was transferred into an NMR tube, and the reaction was monitored by NMR. The NMR spectrometer was pre-set at a specific temperature (50 °C or 80 °C), and NMR spectra were recorded over time to track the appearance of CH_2_-COONH peaks.

#### 3.2.4. General Procedure for the Synthesis of a Polyurethane Thermoset through a Two-Step Method

As an illustrative example, 2.59 g of PMDI or IPDI (2.5 equivalents, 1.17 mol) and 7 mg of DBTDL (0.1 mol%, 0.012 mmol) were placed in a 10 mL round-bottom flask with two necks. The setup was purged with nitrogen for 10 min and then heated to 80 °C. Subsequently, 2.41 g of Velvetol^®^ H500 (1 equivalent, 4.68 mmol) was slowly added using a syringe driver during 1 h. The resulting mixture was stirred magnetically for 4 h at 80 °C. Afterwards, 2 g of the obtained prepolymer was transferred to a PP flask, and 0.174 g of glycerol was added. The mixture was mixed at 2500 rpm for 2 min in a PP flask using a SpeedMixerTM. Next, the mixture was poured into a silicon mold and cured at 80 °C for 24 h in an oven.

## 4. Conclusions

To conclude, p-menthane-1,8-diisocyanate was successfully synthesized using a raw material derived from turpentine, PMDA, through a phosgene-free route at room temperature. The NMR study of PMDA, PMDI, and PMDC has provided, for the first time, a comprehensive understanding of the spatial configuration of the *cis*:*trans* isomers of these three different molecules. Furthermore, we investigated the reaction rate of PMDI with benzyl alcohol in the presence of nine different catalysts to understand their effects. As expected, hard Lewis acid catalysts exhibited the highest reactivity. However, some organic catalysts, such as DBU or TBD, offered a viable alternative to transition-metal catalysts, as their reactivity proved to be quite competitive when compared to tin or bismuth-based catalysts. PMDI was subsequently used in designing a polyurethane material with a bio-based content of 92% that exhibited satisfying physicochemical properties when compared to its petrochemical counterpart. 

We believe that the successful synthesis of PMDI, along with the findings of this research, contributes to the development of more sustainable practices and paves the way for further advancements in environmentally friendly materials.

## Figures and Tables

**Table 3:** Gelation times of IPDI- and PMDI-based formulations.

Gelation Times (s)		
Frequency (Hz)	IPDI-Based Formulation	PMDI-Based Formulation
5 Hz	430	4805
7.1 Hz	424	4933
10 Hz	424	5189
Average	426 ± 3	4975 ± 142

**Table 4:** Thermal and chemical properties of MDI- and BGI-based thermosets.

	$T_{d}^{5\%}$	$T_{d}^{50\%}$	$T_{d}^{85\%}$	*T_g_*	*SI*	*GC*
Unit	(°C)				%	
IPDI-based thermoset	285	348	399	53	50	99
PMDI-based thermoset	240	297	393	18	75	98

## Data Availability

No new data were created, apart from that reported as Supplementary Materials.

## Supplementary Materials

The following supporting information can be downloaded at: https://www.mdpi.com/article/10.3390/molecules28207133/s1.

## Sample Availability

Samples of the compound PMDI are available from the authors.

## References

[1] Polyurethane Market Growth, Size, Global Industry Trend 2032. https://www.factmr.com/report/polyurethane-market.

[2] (2017). Final Report Summary—GRAIL (Glycerol Biorefinery Approach for the Production of High Quality Products of Industrial Value)|FP7|CORDIS|European Commission.

[3] Vaswani S. (2012). Process Economics Program Report 283 Bio-Based 1,4-Butanediol.

[4] Thengumpilil N.B.K., Penumarthy V., Ayyagari A.L. (2002). Process for the Preparation of a Monoglyceride. U.S. Patent.

[5] Mutlu H., Meier M.A.R. (2010). Castor Oil as a Renewable Resource for the Chemical Industry. Eur. J. Lipid Sci. Technol..

[6] Montero De Espinosa L., Meier M.A.R. (2011). Plant Oils: The Perfect Renewable Resource for Polymer Science?!. Eur. Polym. J..

[7] Petrovic Z.S. (2008). Polyurethanes from Vegetable Oils. Polym. Rev..

[8] Peyrton J., Chambaretaud C., Avérous L. (2019). New Insight on the Study of the Kinetic of Biobased Polyurethanes Synthesis Based on Oleo-Chemistry. Molecules.

[9] Gondaliya A., Nejad M. (2021). Lignin as a Partial Polyol Replacement in Polyurethane Flexible Foam. Molecules.

[10] Li H., Sun J.-T., Wang C., Liu S., Yuan D., Zhou X., Tan J., Stubbs L., He C. (2017). High Modulus, Strength, and Toughness Polyurethane Elastomer Based on Unmodified Lignin. ACS Sustain. Chem. Eng..

[11] Sternberg J., Sequerth O., Pilla S. (2021). Green Chemistry Design in Polymers Derived from Lignin: Review and Perspective. Prog. Polym. Sci..

[12] Henry C., Gondaliya A., Thies M., Nejad M. (2022). Studying the Suitability of Nineteen Lignins as Partial Polyol Replacement in Rigid Polyurethane/Polyisocyanurate Foam. Molecules.

[13] Tomaselli S., Bertini F., Cifarelli A., Vignali A., Ragona L., Losio S. (2023). Antibacterial Properties of Polyurethane Foams Additivated with Terpenes from a Bio-Based Polyol. Molecules.

[14] Debuissy T., Sangwan P., Pollet E., Avérous L. (2017). Study on the Structure-Properties Relationship of Biodegradable and Biobased Aliphatic Copolyesters Based on 1,3-Propanediol, 1,4-Butanediol, Succinic and Adipic Acids. Polymer.

[15] Debuissy T., Pollet E., Avérous L. (2017). Synthesis and Characterization of Fully Biobased Poly(Propylene Succinate-Ran-Propylene Adipate). Analysis of the Architecture-Dependent Physicochemical Behavior. J. Polym. Sci. A Polym. Chem..

[16] Kluge M., Pérocheau Arnaud S., Robert T. (2019). 1,3-Propanediol and Its Application in Bio-Based Polyesters for Resin Applications. Chem. Afr..

[17] Sadavarte Nilakshi V. (2012). Difunctional Monomers Starting from Cashew Nut Shell Liquid (CNSL) and High Performance Polymers Therefrom. Ph.D. Thesis.

[18] Chatterjee D., Sadavarte N.V., Shingte R.D., More A.S., Tawade B.V., Kulkarni A.D., Ichake A.B., Avadhani C.V., Wadgaonkar P.P. (2017). Step-Growth Polymers from Cashew Nut Shell Liquid (CNSL)-Based Aromatic Difunctional Monomers. Cashew Nut Shell Liquid.

[19] Kuhire S.S., Ichake A.B., Grau E., Cramail H., Wadgaonkar P.P. (2018). Synthesis and Characterization of Partially Bio-Based Polyimides Based on Biphenylene-Containing Diisocyanate Derived from Vanillic Acid. Eur. Polym. J..

[20] Neumann C.N.D., Bulach W.D., Rehahn M., Klein R. (2011). Water-Free Synthesis of Polyurethane Foams Using Highly Reactive Diisocyanates Derived from 5-Hydroxymethylfurfural. Macromol. Rapid Commun..

[21] Ghosh A.K., Sarkar A., Brindisi M. (2018). The Curtius Rearrangement: Mechanistic Insight and Recent Applications in Natural Product Syntheses. Org. Biomol. Chem..

[22] Lemouzy S., Delavarde A., Lamaty F., Bantreil X., Pinaud J., Caillol S. (2023). Lignin-Based Bisguaiacol Diisocyanate: A Green Route for the Synthesis of Biobased Polyurethanes. Green Chem. J..

[23] Knölker H.-J., Braxmeier T., Schlechtingen G. (1995). A Novel Method for the Synthesis of Isocyanates Under Mild Conditions. Angew. Chem. Int. Ed. Engl..

[24] Huang D., Zhu S., Lan H., Lin Z., Wang X. (2019). Design, Synthesis and Herbicidal Activities of (3R,4R)-4,7,7-Trimethyl-6-Oxabicyclo[3.2.1]Octane-3,4-Diol Derivatives. Ind. Crops Prod..

[25] Zhu S., Xu S., Yi X., Wang J., Zhao Z., Jiang J. (2018). High Value-Added Application of Turpentine as a Potential Renewable Source for the Synthesis of Heterocyclic Schiff Base Derivatives of Cis-1,8-p-Menthane-Diamine Serving as Botanical Herbicides. Ind. Crops Prod..

[26] Yi X., Xu S., Zhao Z. (2015). Progress on Preparation and Application of P-Menthane-1,8-Diol Monohydrate. Proceedings of the 3rd International Conference on Material, Mechanical and Manufacturing Engineering.

[27] Kovals’skaya S.S., Kozlov N.G., Tikhonova T.S. (1989). Stereoselective Synthesis of *N*,*N*′-Diacyl-p-Menthane-1,8-Diamines. Chem. Nat. Compd..

[28] Dalling D.K., Grant D.M. (1972). Carbon-13 Magnetic Resonance. XXI. Steric Interactions in the Methylcyclohexanes. J. Am. Chem. Soc..

[29] Kapp R.W. (2014). Isocyanates. Encyclopedia of Toxicology.

[30] Kwon J.-Y., Yoo H.-J., Kim H.-D. (2001). Effect of Chemical Structure on the Properties of UV-Cured Polyurethane Acrylates Films. Fibers Polym..

[31] Zhang Y., Xia Z., Huang H., Chen H. (2009). Thermal Degradation of Polyurethane Based on IPDI. J. Anal. Appl. Pyrolysis.

[32] Akram N., Zia K.M., Saeed M., Usman M., Khan W.G. (2019). Role of Isophorone Diisocyanate in the Optimization of Adhesion Tendency of Polyurethane Pressure Sensitive Adhesives. J. Appl. Polym. Sci..

[33] Dasgupta A., van Ingen Y., Guerzoni M.G., Farshadfar K., Rawson J.M., Richards E., Ariafard A., Melen R.L. (2022). Lewis Acid Assisted Brønsted Acid Catalysed Decarbonylation of Isocyanates: A Combined DFT and Experimental Study. Chem. A Eur. J..

[34] Lhomme J. (2013). Nouveaux Catalyseurs et Systèmes Catalytiques Appliqués à La Synthèse Du Polyuréthane via La Réaction Isocyanate—Alcool. Ph.D. Thesis.

[35] Schwetlick K., Noack R. (1995). Kinetics and Catalysis of Consecutive Isocyanate Reactions. Formation of Carbamates, Allophanates and Isocyanurates. J. Chem. Soc. Perkin Trans..

[36] Luo S.-G., Tan H.-M., Zhang J.-G., Wu Y.-J., Pei F.-K., Meng X.-H. (1997). Catalytic Mechanisms of Triphenyl Bismuth, Dibutyltin Dilaurate, and Their Combination in Polyurethane-Forming Reaction. J. Appl. Polym. Sci..

[37] Van Maris R., Tamano Y., Yoshimura H., Gay K.M. (2005). Polyurethane Catalysis by Tertiary Amines. J. Cell. Plast..

[38] Thiele L., Becker R. (1993). Catalytic Mechanisms of Polyurethane Formation. Adv. Urethane Sci. Technol..

[39] Alsarraf J., Ammar Y.A., Robert F., Cloutet E., Cramail H., Landais Y. (2012). Cyclic Guanidines as Efficient Organocatalysts for the Synthesis of Polyurethanes. Macromolecules.

[40] Arnould P., Simon F., Fouquay S., Pardal F., Michaud G., Gajan D., Raynaud J., Monteil V. (2022). Harnessing Catalysis Selectivity and Isophorone Diisocyanate Asymmetry for Tailored Polyurethane Prepolymers and Networks. Macromolecules.

[41] Schwetlick K., Noack R., Stebner F. (1994). Three Fundamental Mechanisms of Base-Catalysed Reactions of Isocyanates with Hydrogen-Acidic Compounds. J. Chem. Soc. Perkin Trans..

[42] Lomölder R., Plogmann F., Speier P. (1997). Selectivity of Isophorone Diisocyanate in the Urethane Reaction Influence of Temperature, Catalysis, and Reaction Partners. J. Coat. Technol..

[43] Gerard J.-F., Perchec P.L., Pham Q.T. (1988). Polyuréthannes à Propriétés Emulsifiantes et Électrolytiques, 2. Cinétique de Polycondensation En Solution Des Alkylimino-2,2′ Diéthanols Avec Le Diisocyanate d’isophorone. Etude Par 1H et 13C NMR. Die Makromol. Chem..

[44] Cunliffe A.V., Davis A., Farey M., Wright J. (1985). The Kinetics of the Reaction of Isophorone Di-Isocyanate with Mono-Alcohols. Polymer.

[45] Lorenz O., Decker H., Rose G. (1984). NCO-Prepolymere Aus Diisocyanaten Mit Unterschiedlich Reaktiven NCO-Gruppen. Angew. Makromol. Chem..

[46] Hatada K., Ute K., Peter Pappas S. (1987). *E*,*Z* Assignments of Isophorone Diisocyanate (IPDI) and Their Implications on the Relative Reactivity of the Isocyanate Groups. J. Polym. Sci. Part C Polym. Lett..

[47] Bialas N., Höcker H., Marschner M., Ritter W. (1990). 13C NMR Studies on the Relative Reactivity of Isocyanate Groups of Isophorone Diisocyanate Isomers. Die Makromol. Chem..

[48] Blank W.J., He Z.A., Hessell E.T. (1999). Catalysis of the Isocyanate-Hydroxyl Reaction by Non-Tin Catalysts. Prog. Org. Coat..

