# Supplementary material for: Paving the Way towards Sustainability of Polyurethanes: Synthesis and Properties of Terpene-Based Diisocyanate
Source: Molecules. 2023 Oct 17;28(20):7133. doi: 10.3390/molecules28207133 (PMC10609554; doi:10.3390/molecules28207133)
Supplement: Supplementary file 1 [file molecules-28-07133-s001.zip › molecules-2658000-supplementary.docx]

*Supplementary Information*

Paving the Way towards Sustainability of Polyurethanes: Synthesis and Properties of Terpene-Based Diisocyanate

Aliénor Delavarde ^1^, Sebastien Lemouzy ^1^, Aurélien Lebrun ^2^, Julien Pinaud ^1^ and Sylvain Caillol ^1,^*

^1^ ICGM, Univ Montpellier, CNRS, ENSCM, 34095 Montpellier, France; alienor.delavarde@enscm.fr (A.D.); sebastien.lemouzy@enscm.fr (S.L.); julien.pinaud@umontpellier.fr (J.P.)

^2^ IBMM, Univ Montpellier, CNRS, ENSCM, 34095 Montpellier, France;
aurelien.lebrun@umontpellier.fr

* Correspondence: sylvain.caillol@enscm.fr

1. **Characterization of the products and the polyurethane materials**
   1. **P-menthane-1,8-diamine (PMDA) characterizations**

**Figure S1** Molecular structure assignment of PMDA

**Figure S2** ^1^H NMR spectrum of PMDA (in CDCl_3_) on 600 MHz spectrometer

**Figure S3** ^13^C NMR spectrum of PMDA (in CDCl_3_) on 600 MHz spectrometer

**
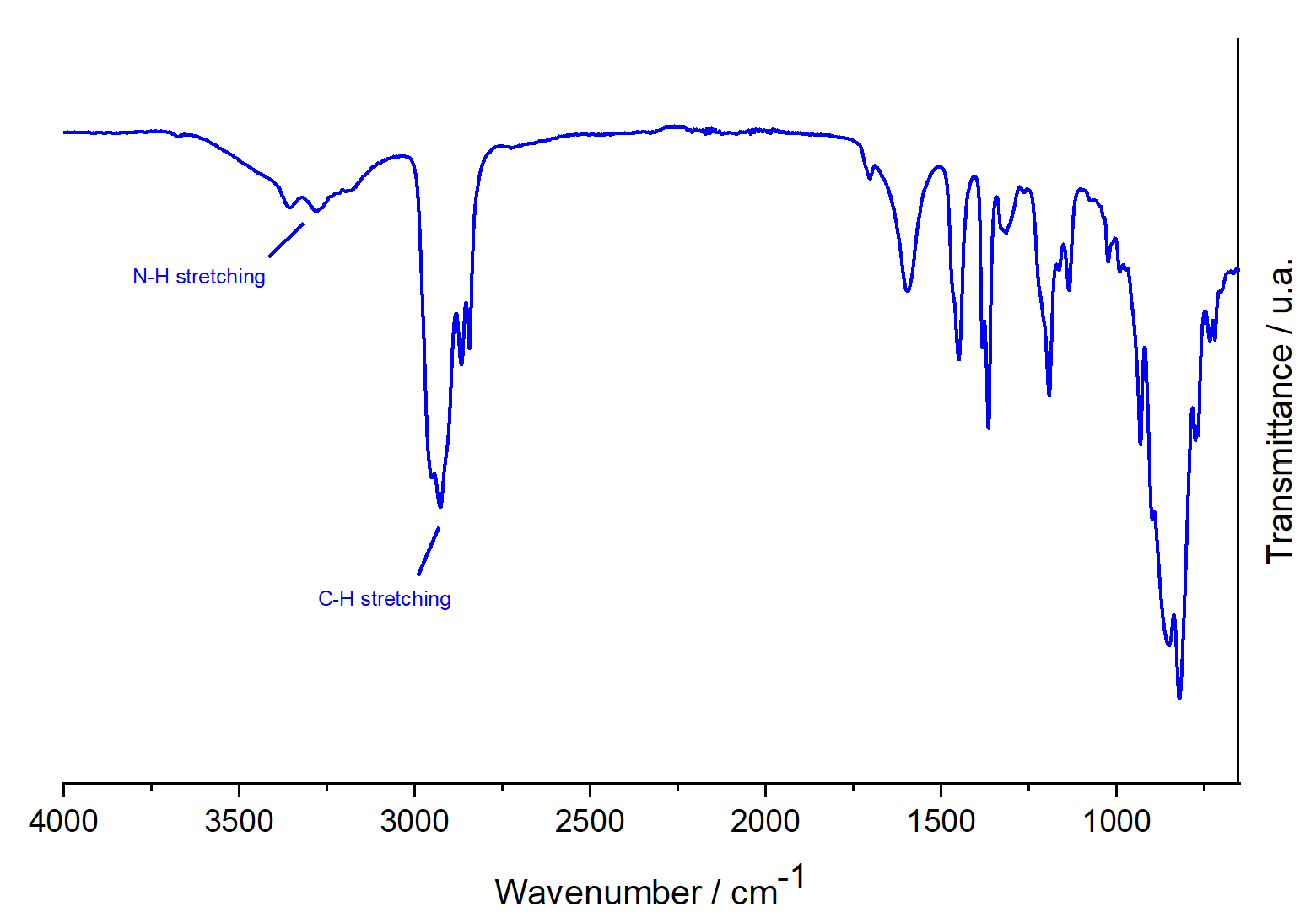
**

**Figure S4** FTIR spectrum of PMDA

**
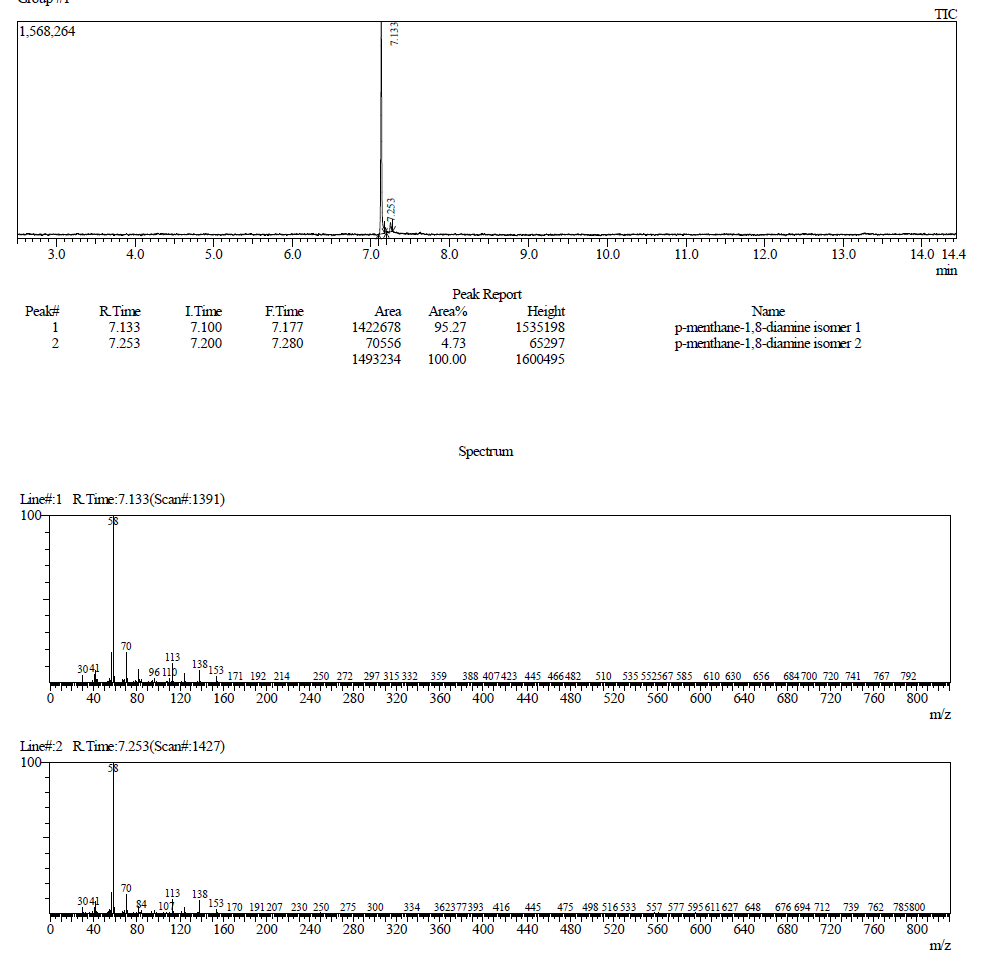
**

**Figure S5** (GC)-MS of PMDA

- 1. **P-menthane-1,8-diisocyanate (PMDI) characterizations**

**Figure S6** Molecular structure assignment of PMDI

**Figure S7** ^1^H NMR spectrum of PMDI (in CDCl_3_) on 600 MHz spectrometer

**Figure S8** ^13^C NMR spectrum of PMDI (in CDCl_3_) on 600 MHz spectrometer


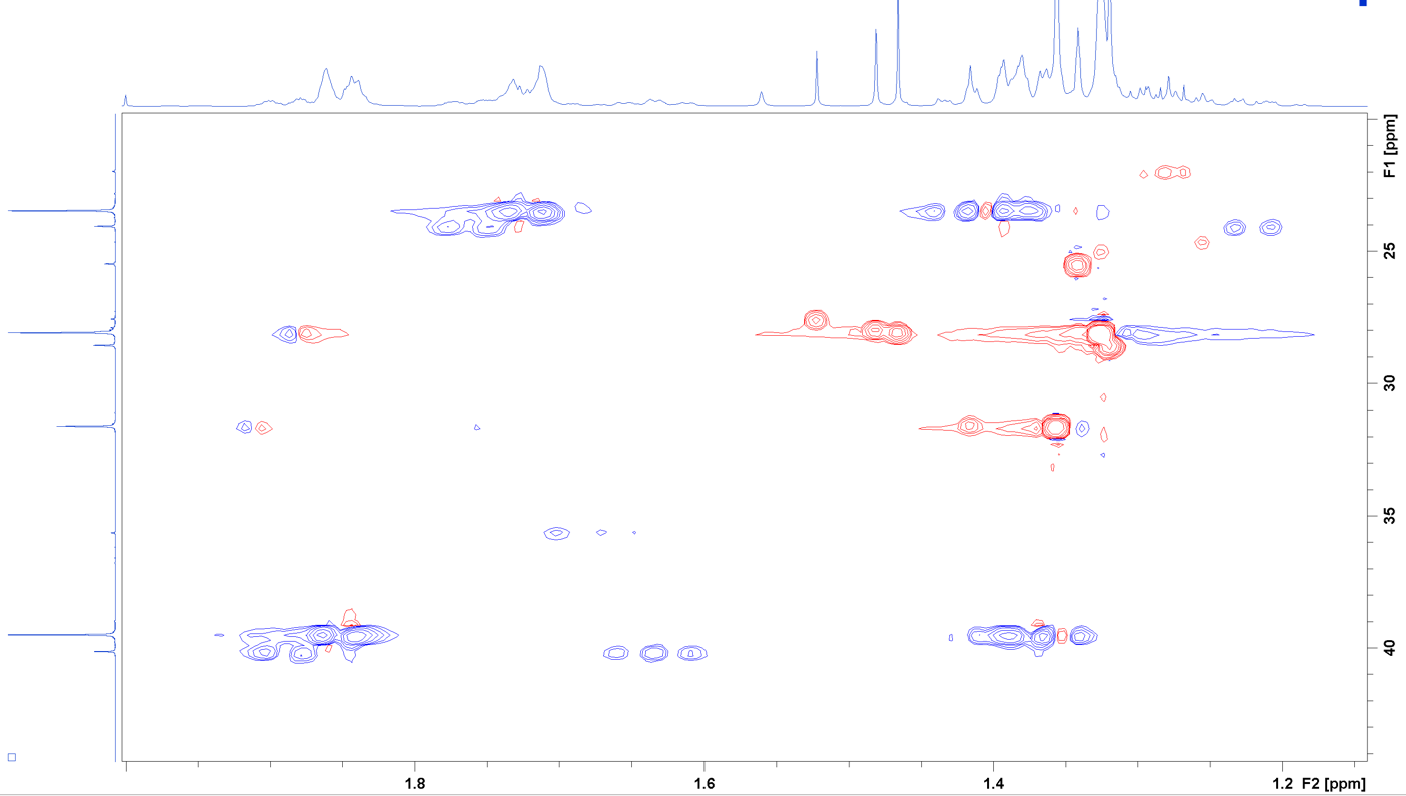


**Figure S9** ^13^C-^1^H HSQC spectrum of PMDI (in CDCl_3_) on 600 MHz spectrometer


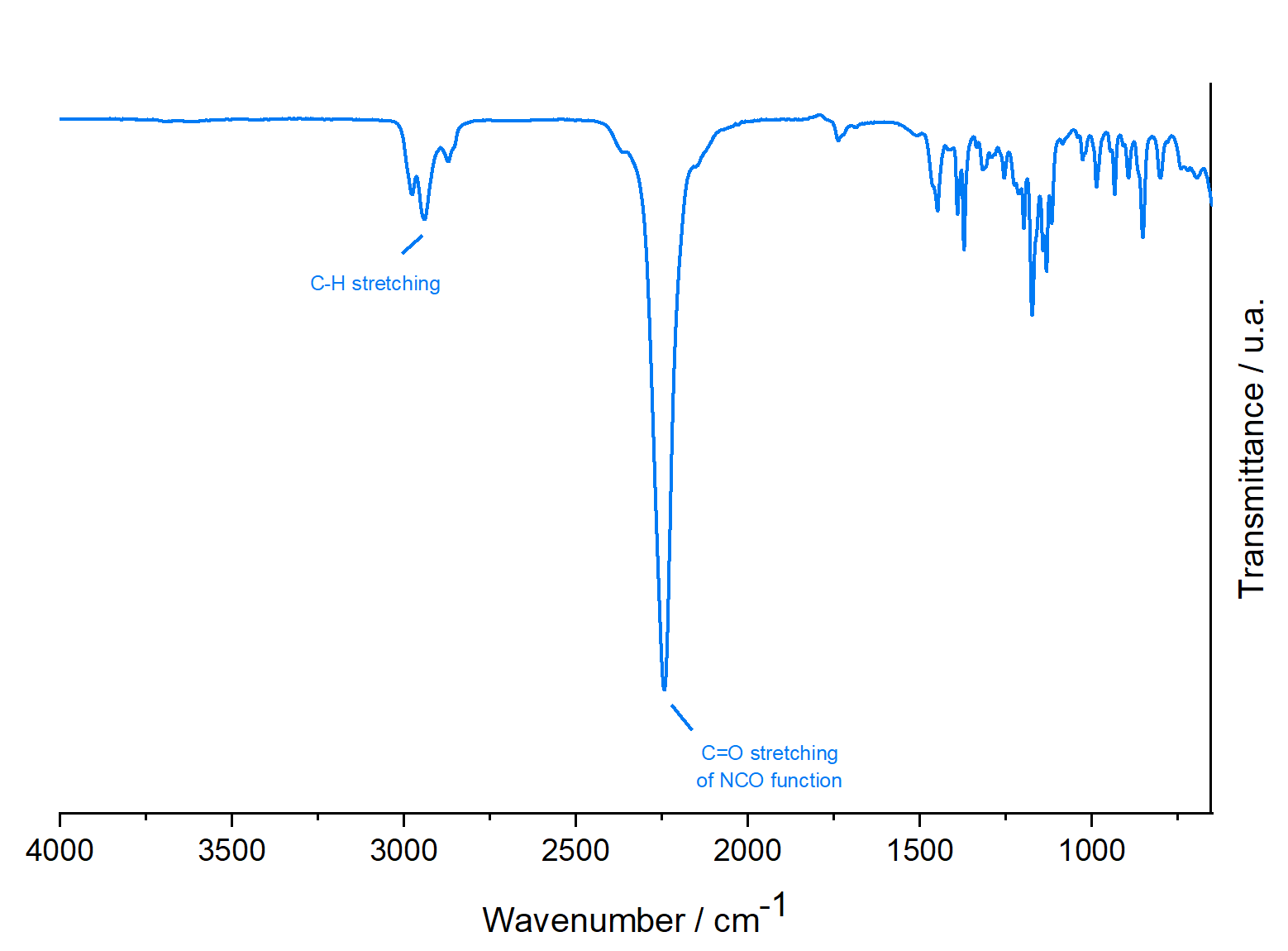


**Figure S10** FTIR spectrum of PMDI


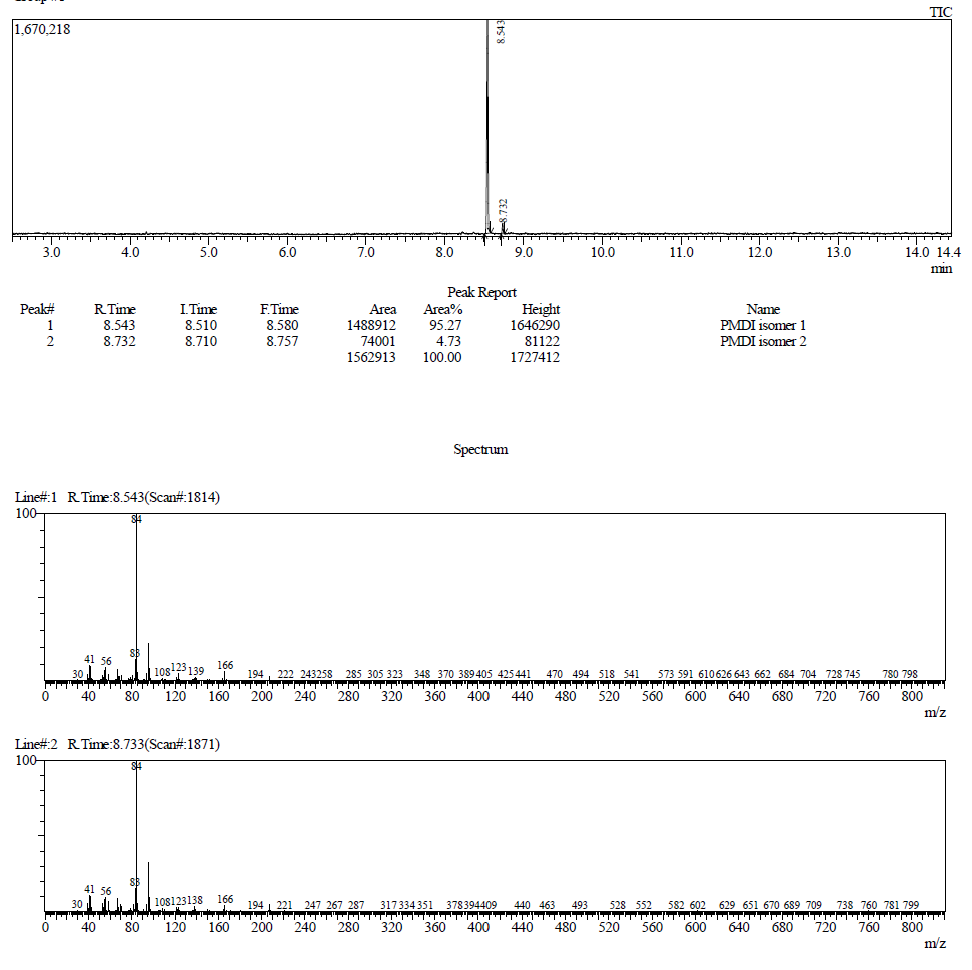


**Figure S11** (GC)-MS of PMDI

- 1. **P-menthane-1,8-dicarbamate (PMDC) characterizations**

**Figure S12** Molecular structure assignment of PMDC

**Figure S13** ^1^H NMR spectrum of PMDC (in CDCl_3_) on 400 MHz spectrometer

**Figure S14** ^13^C NMR spectrum of PMDC (in CDCl_3_) on 400 MHz spectrometer


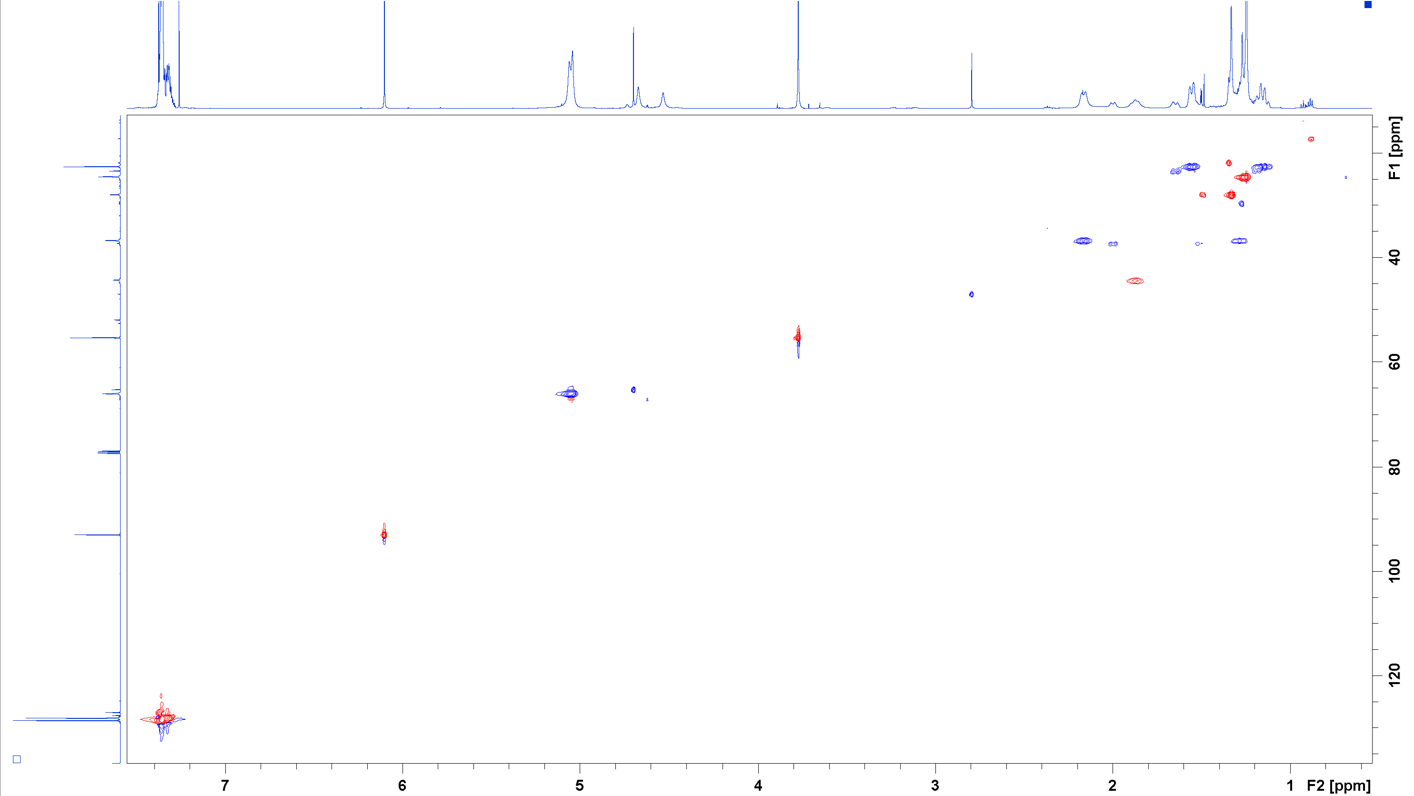


**Figure S15** ^13^C-^1^H HSQC spectrum of PMDC (in toluene-d_8_) on 600 MHz spectrometer


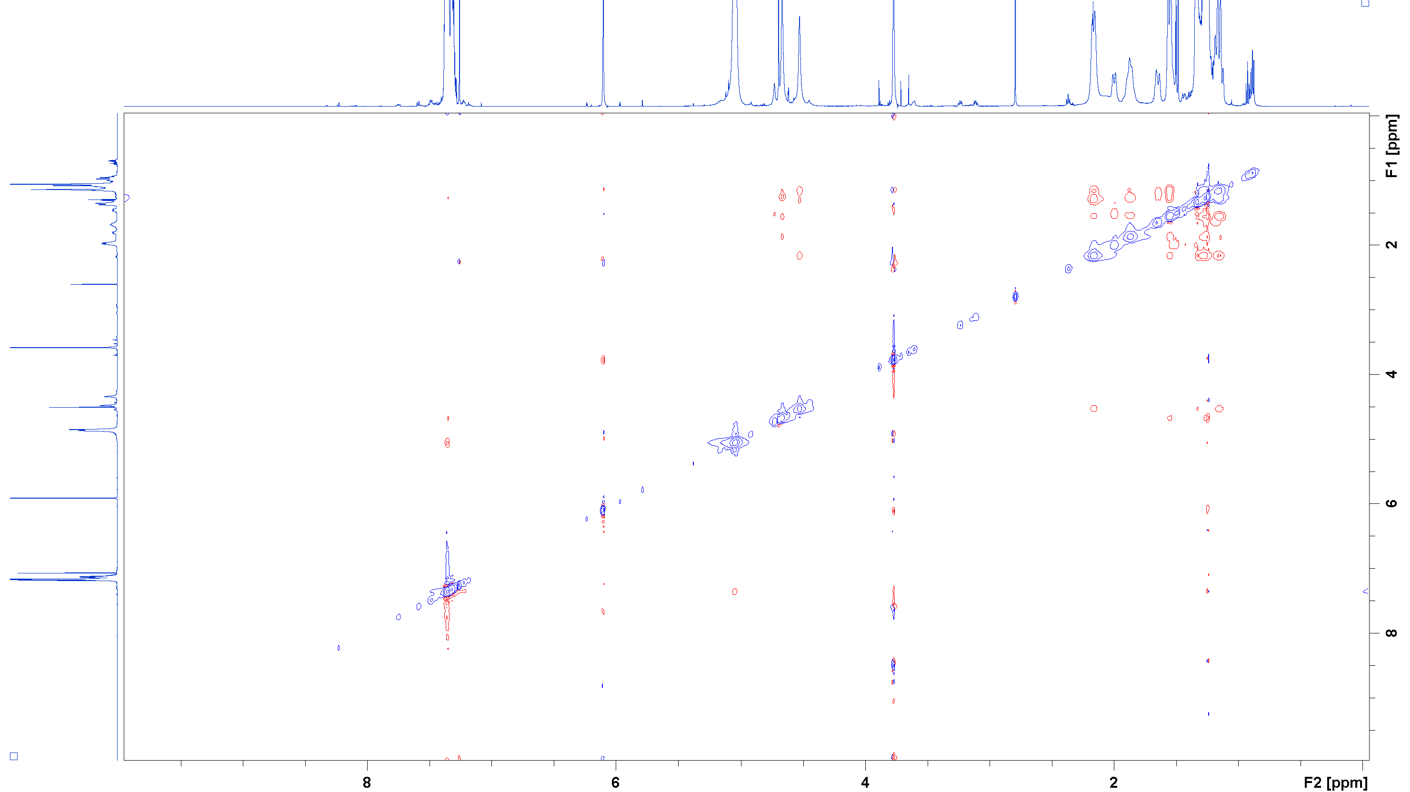


**Figure S16** NOESY spectrum of PMDC (in toluene-d_8_) on 600 MHz spectrometer


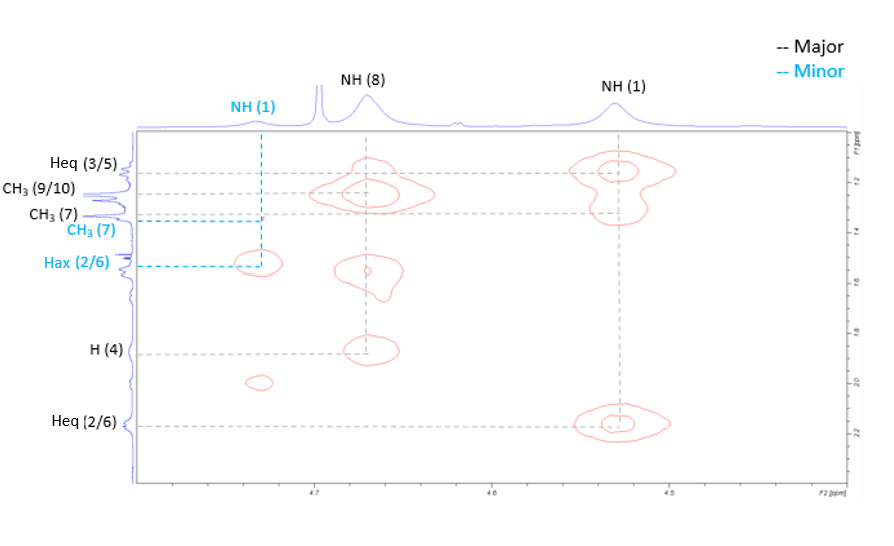


**Figure S17** NOE correlations of PMDC (in toluene-d_8_ on 600 MHz spectrometer) used to determine the position of 1

- 1. **IPDI-based prepolymer characterization**

**Figure S18** Molecular structure assignment of IPDI-based prepolymer

**Figure S19** ^13^C NMR spectrum of IPDI-based prepolymer (in CDCl_3_) on 400 MHz spectrometer


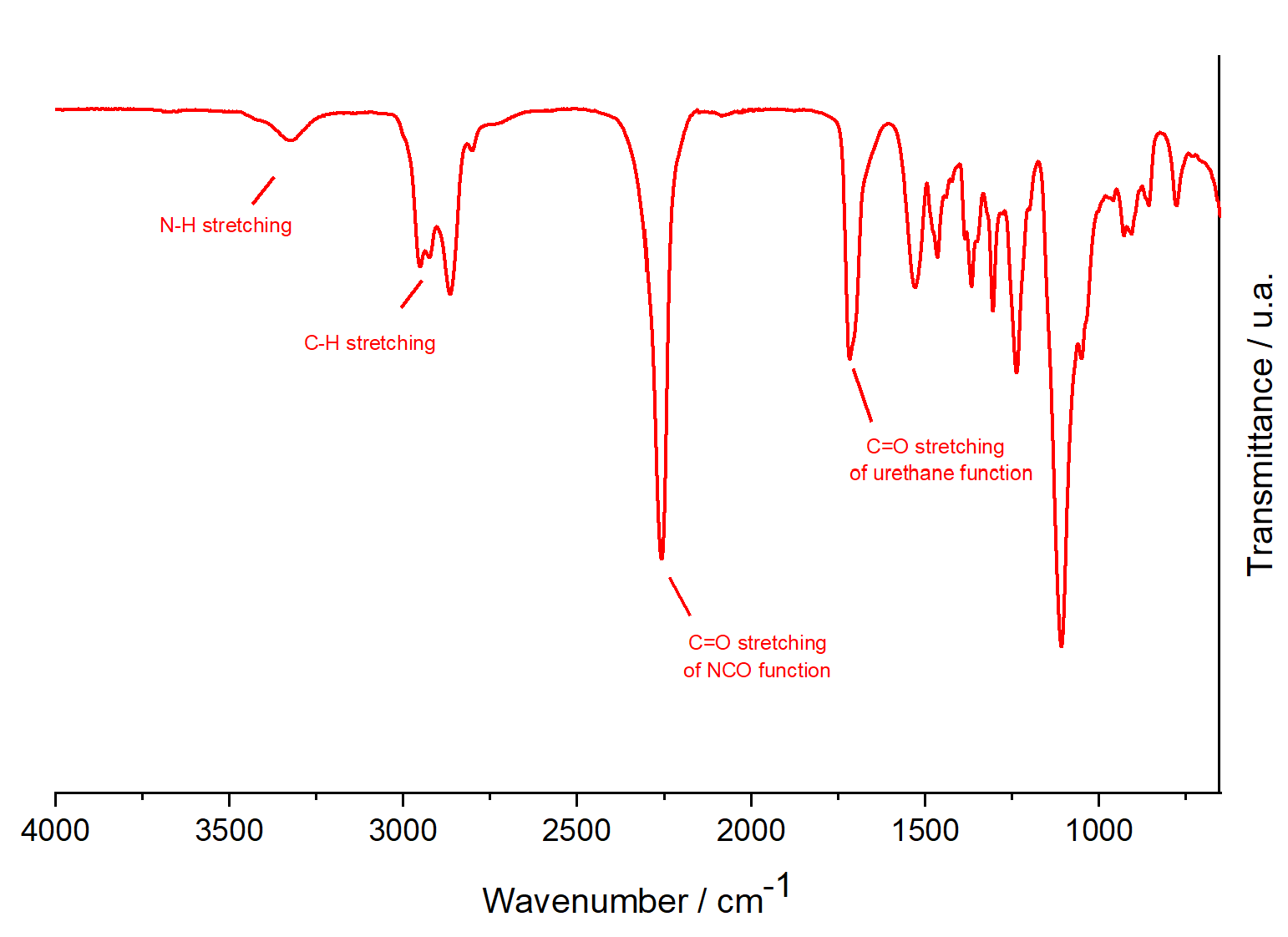


**Figure S20** FTIR spectrum of IPDI-based prepolymer

- 1. **PMDI-based prepolymer characterization**

**Figure S21** Molecular structure assignment of PMDI-based prepolymer

**Figure S22** ^13^C NMR spectrum of PMDI-based prepolymer (in CDCl_3_) on 400 MHz spectrometer


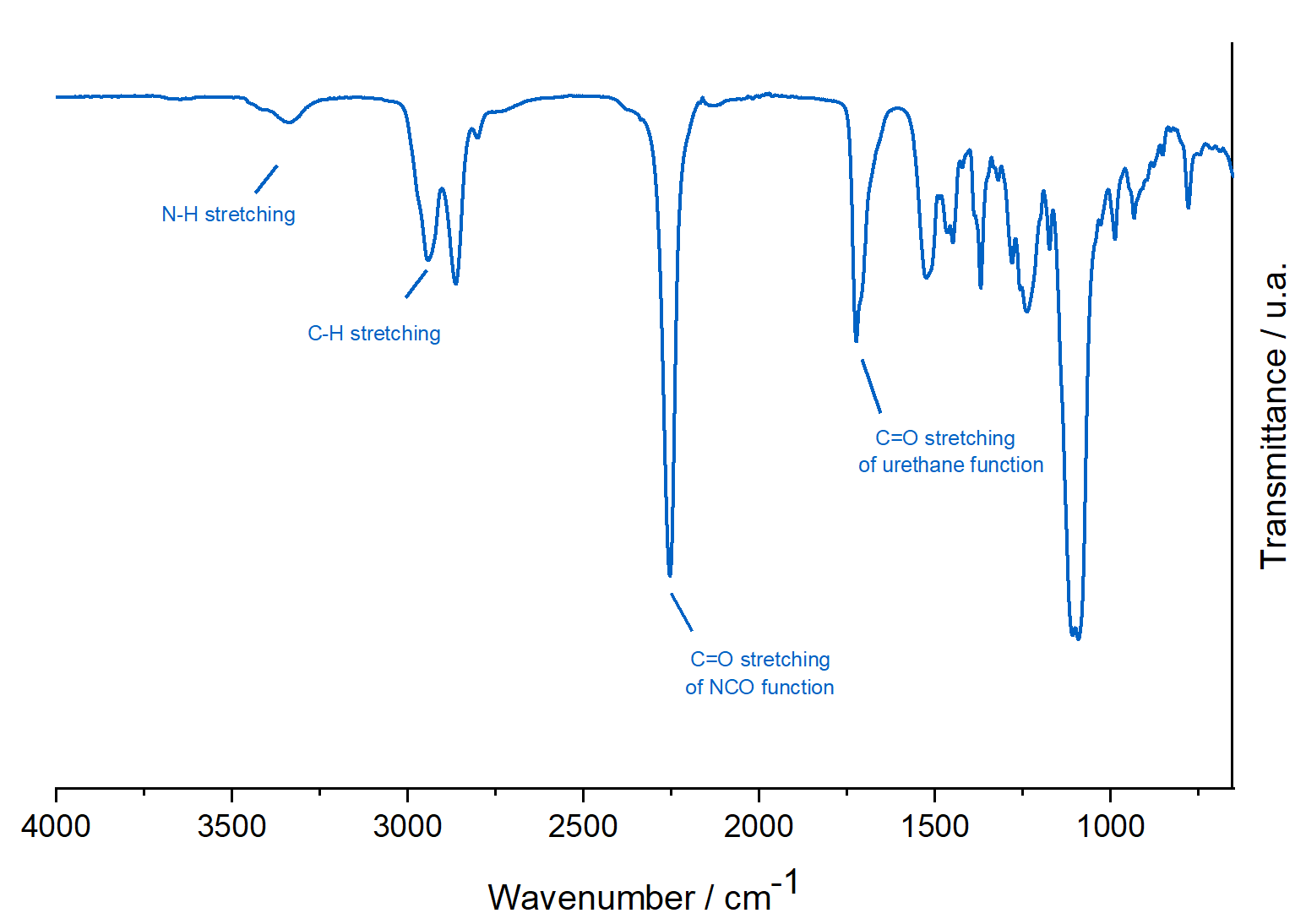


**Figure S23** FTIR spectrum of PMDI-based prepolymer

- 1. **IPDI-based cured material characterizations**


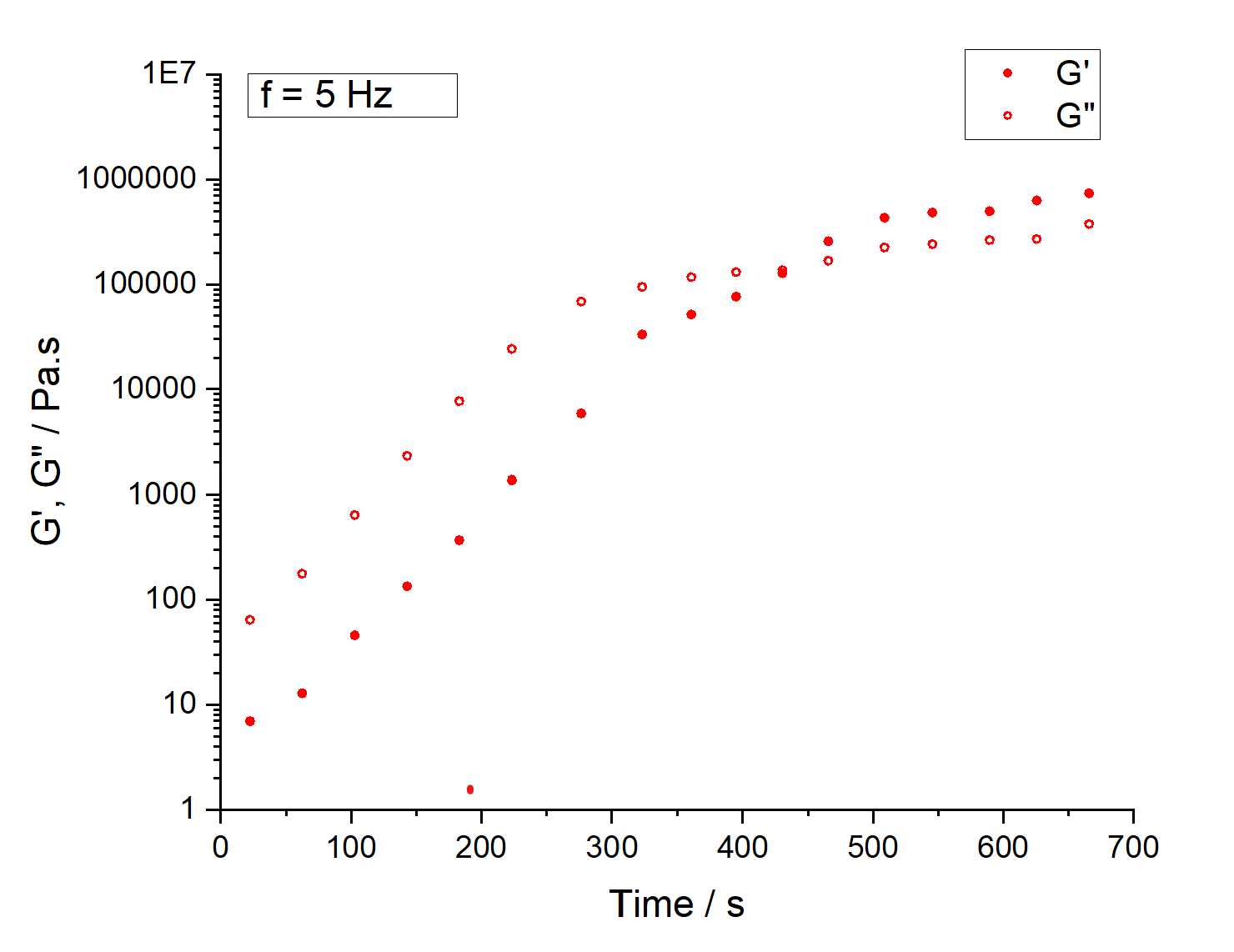

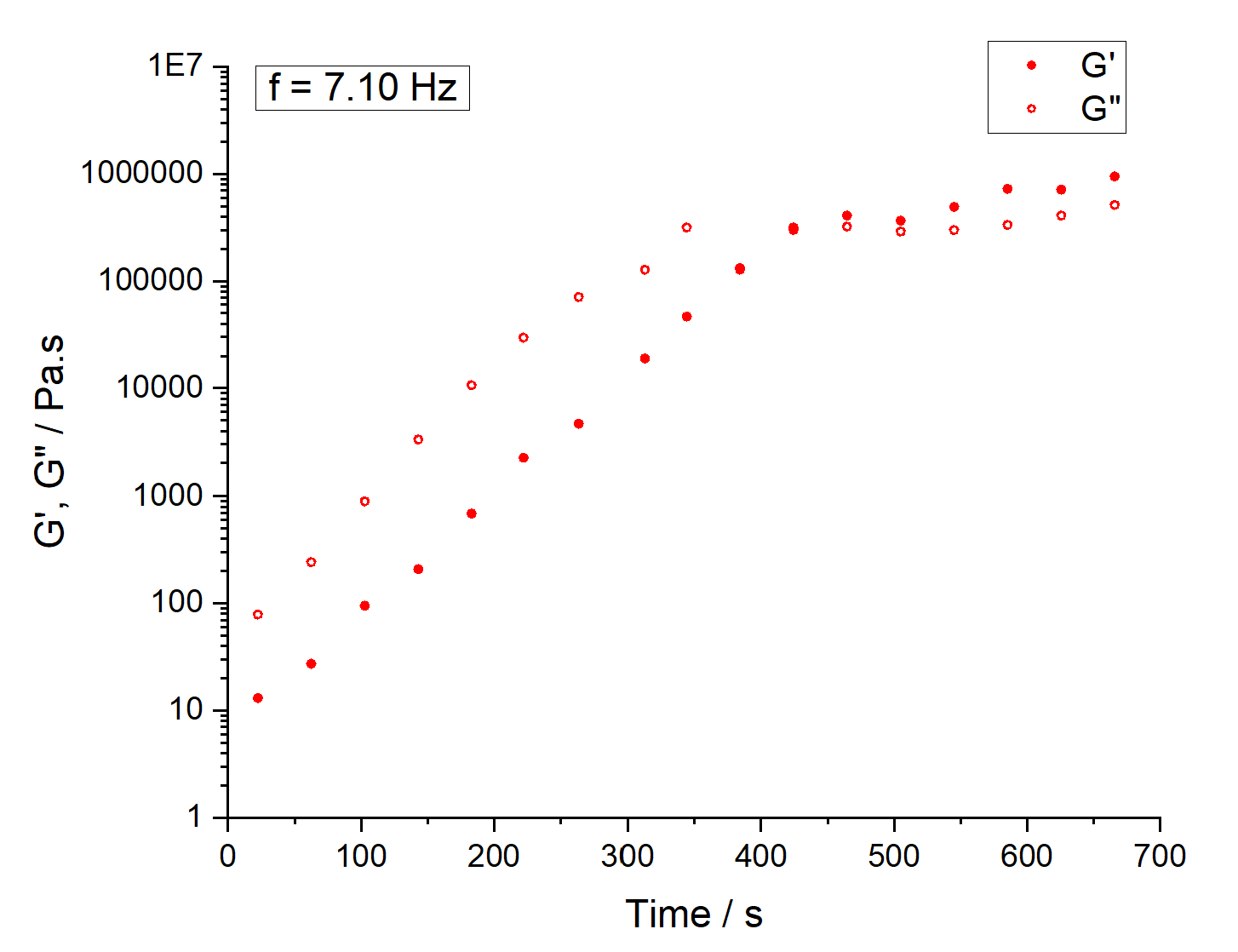


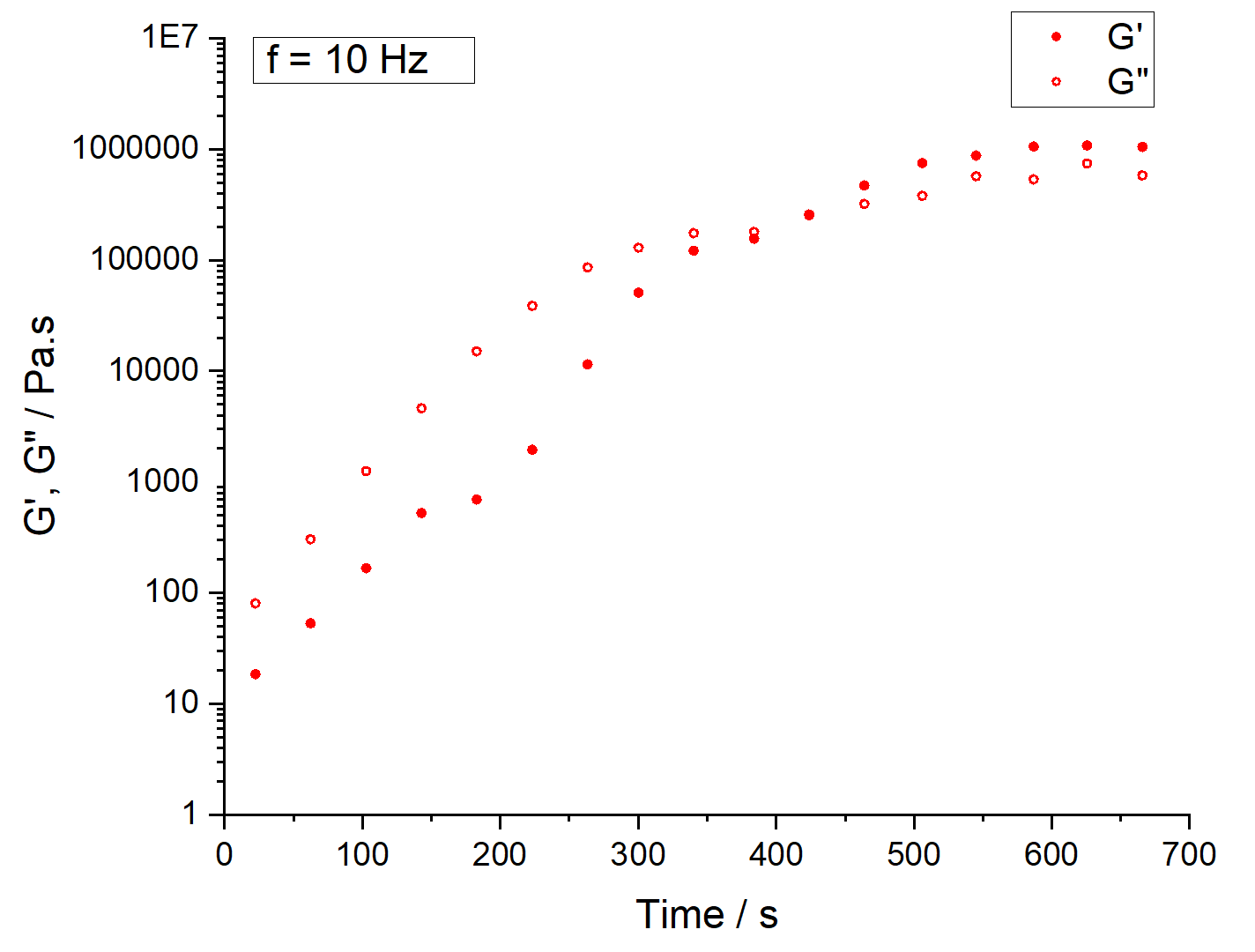


**Figure S24** Gelation time determination of IPDI-based formulation at 80°C (f=5 Hz, 7.1 Hz, 10 Hz)


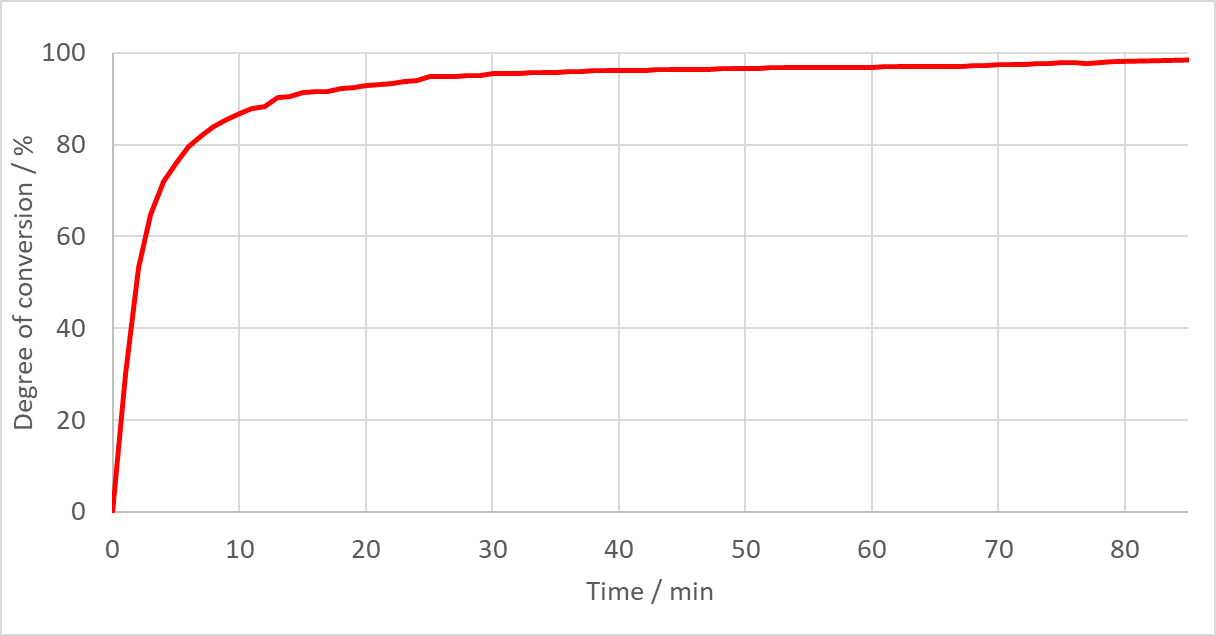


**Figure S25** Curing conversion of IPDI-based formulation monitoring by FTIR spectroscopy at 80 °C

- 1. **PMDI-based cured material characterizations**


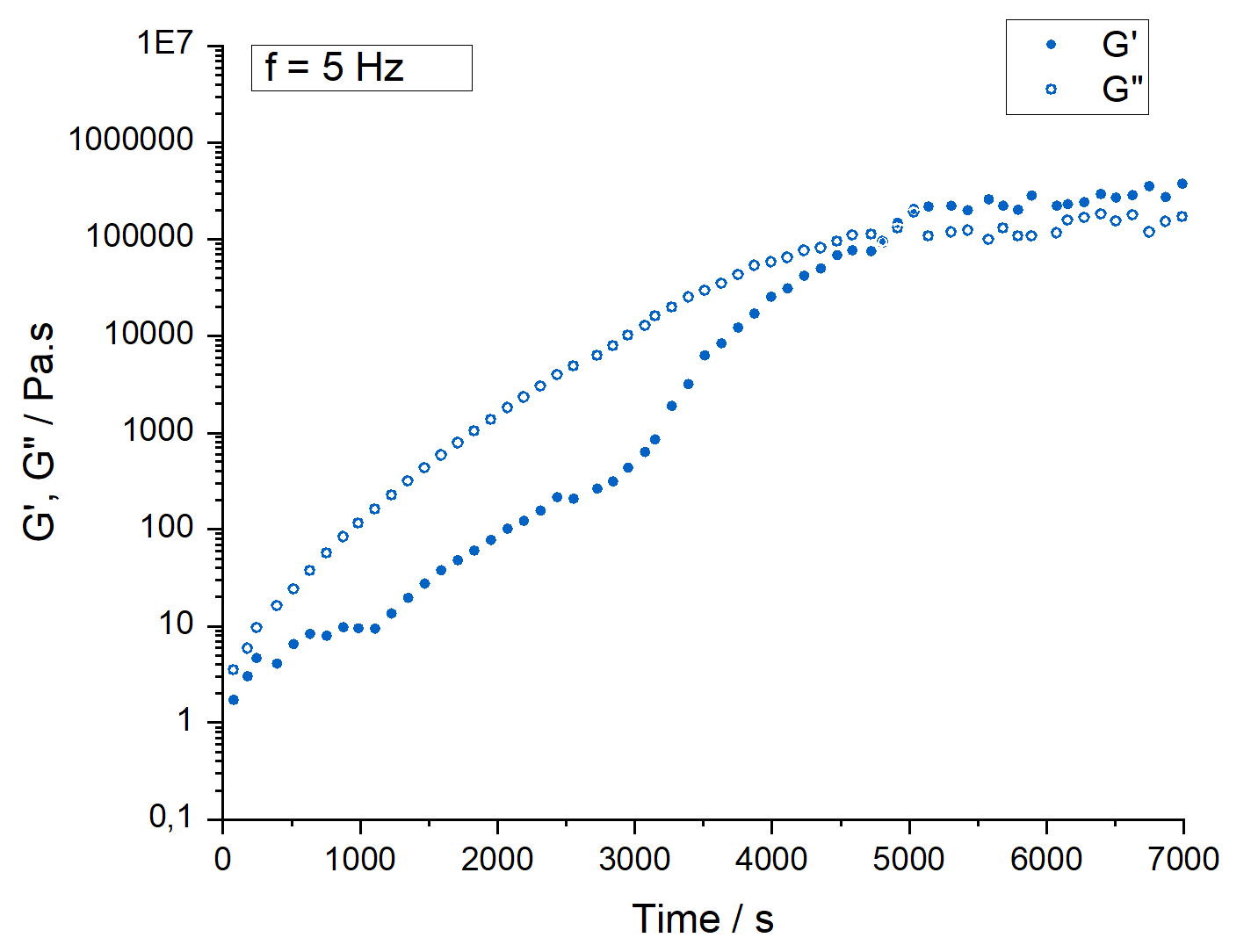

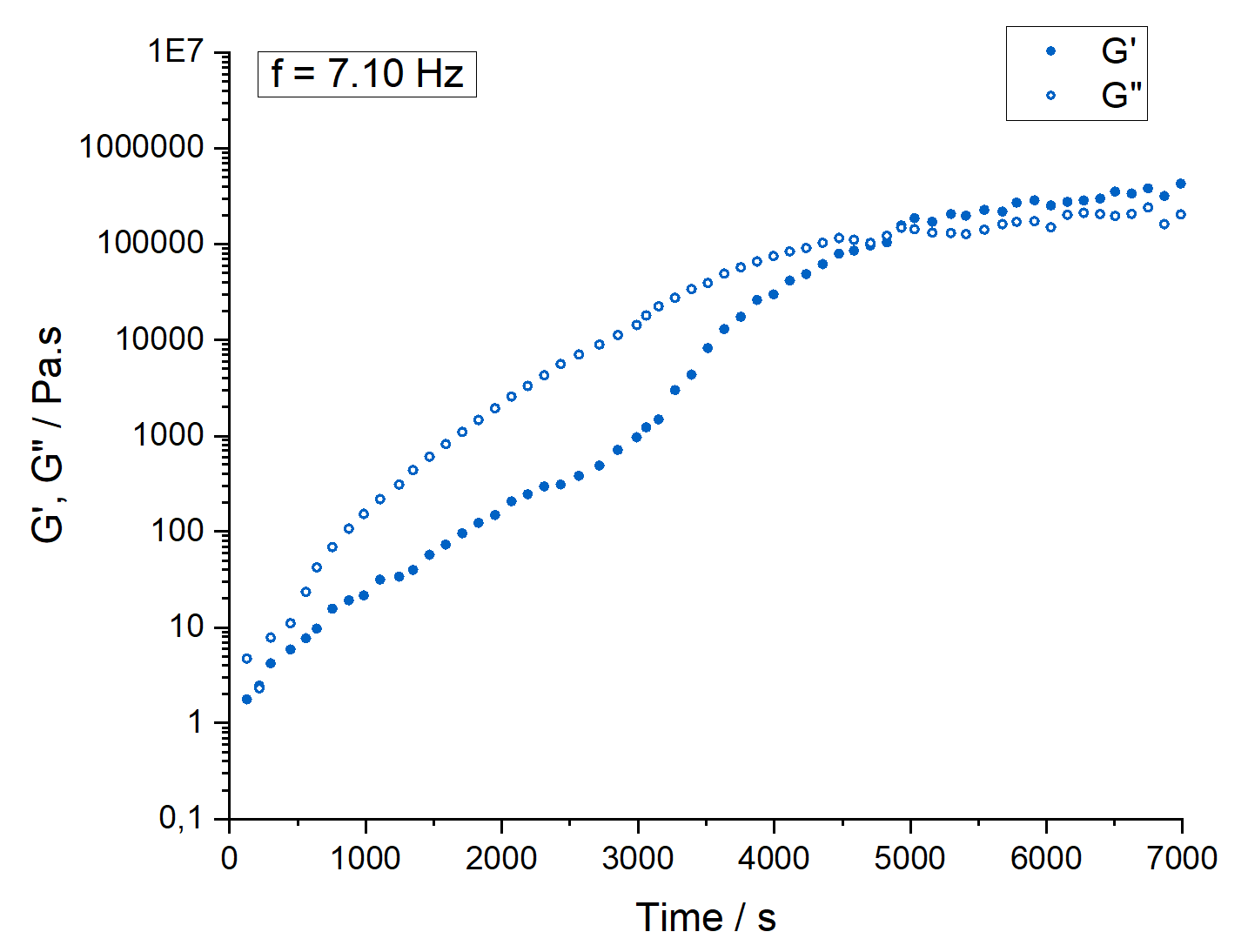


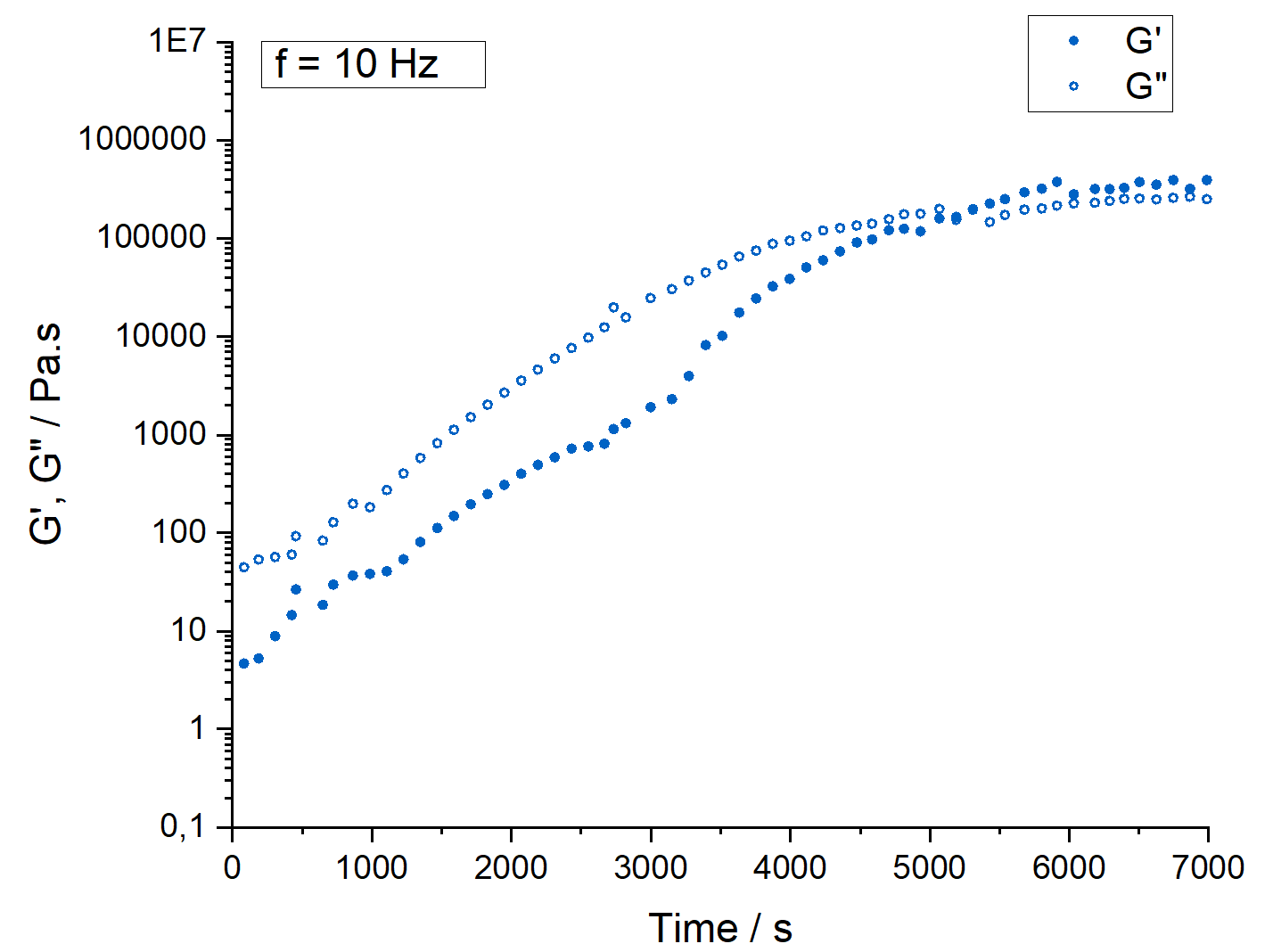


**Figure S26** Gelation time determination of PMDI-based formulation at 80°C (f=5 Hz, 7.1 Hz, 10 Hz)


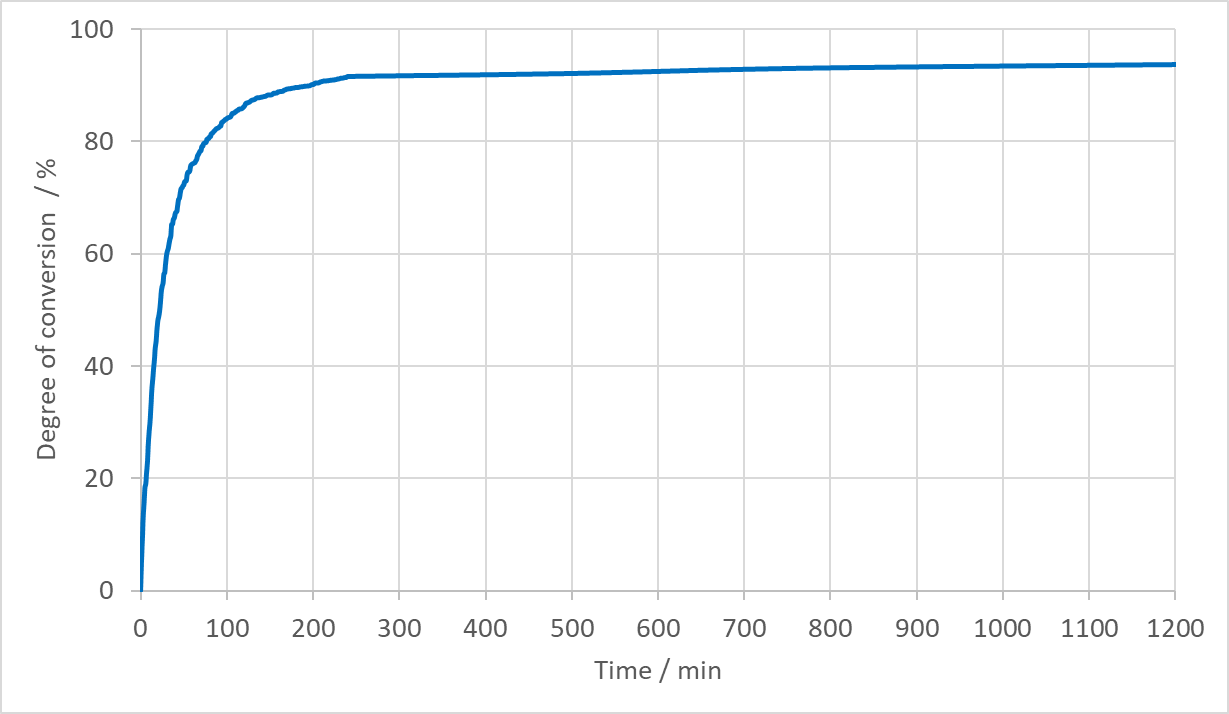


**Figure S27** Curing conversion of PMDI-based formulation monitoring by FTIR spectroscopy at 80 °C
